# Neuroimaging meta regression for coordinate based meta analysis data with a spatial model

**DOI:** 10.1093/biostatistics/kxae024

**Published:** 2024-07-13

**Authors:** Yifan Yu, Rosario Pintos Lobo, Michael Cody Riedel, Katherine Bottenhorn, Angela R Laird, Thomas E Nichols

**Affiliations:** Oxford Big Data Institute, University of Oxford, Old road campus, Oxford, OX3 7LF, United Kingdom; Department of Psychology, Florida International University, Miami, FL, 33199, United States; Department of Physics, Florida International University, Miami, FL, 33199, United States; Department of Population and Public Health Sciences, University of Southern California, Los Angeles, CA, 90032, United States; Center for Imaging Science, Florida International University, Miami, FL, 33199, United States; Oxford Big Data Institute, University of Oxford, Old road campus, Oxford, OX3 7LF, United Kingdom; Nuffield Department of Clinical Neurosciences, Wellcome Centre for Integrative Neuroimaging, FMRIB, Oxford, OX3 9DU, United Kingdom

## Abstract

Coordinate-based meta-analysis combines evidence from a collection of neuroimaging studies to estimate brain activation. In such analyses, a key practical challenge is to find a computationally efficient approach with good statistical interpretability to model the locations of activation foci. In this article, we propose a generative coordinate-based meta-regression (CBMR) framework to approximate a smooth activation intensity function and investigate the effect of study-level covariates (e.g. year of publication, sample size). We employ a spline parameterization to model the spatial structure of brain activation and consider four stochastic models for modeling the random variation in foci. To examine the validity of CBMR, we estimate brain activation on 20 meta-analytic datasets, conduct spatial homogeneity tests at the voxel level, and compare the results to those generated by existing kernel-based and model-based approaches. Keywords: generalized linear models; meta-analysis; spatial statistics; statistical modeling.

## 1 Introduction

Functional neuroimaging includes a number of techniques to image brain activity, including positron emission tomography (PET) and functional magnetic resonance imaging (fMRI). Starting three decades ago, PET studies were used to compare brain activity between rest and experimental conditions, producing maps of “activation,” images of statistics measuring the strength of the experimental effect. Especially in the last two decades, the literature on fMRI activations has grown rapidly, which motivates a need to integrate findings, establish consistency and explore heterogeneity across independent but related studies. However in both PET and fMRI studies, validity is challenged by common drawbacks such as small sample sizes, a high prevalence of false positives (approximately 10−20% of reported foci in publications are false positives [Wager et al. 2007]), significant heterogeneity among studies and unreliable inference due to their diversity in measurements and types of analysis (Samartsidis et al. 2017). Meta-analysis is an essential tool to address these limitations and improve statistical power by pooling evidence from multiple studies and providing insight into consistent results. While there are also applications of neuroimaging meta-analysis to resting-state fMRI and structural analysis using voxel-based morphometry, in this work we focus on fMRI but note that our work applies to data from other types of studies.

Meta-analysis in neuroimaging research is classified into two categories: image-based meta-analysis (IBMA) which uses the 3D statistical maps of original studies and coordinate-based meta-analysis (CBMA) which uses the reported spatial coordinates of activation foci in standard MNI or Talairach space. Ideally, only IBMA would be used, as there is substantial information loss by only using activation foci as compared to full statistics maps, and further accuracy loss occurs when deactivation foci are ignored (Salimi-Khorshidi et al. 2009). However, while it is now more common to share entire statistical maps in published studies, historically, researchers typically reported only the *x*, *y*, *z* coordinates of peak activation (local maxima) within each activation region. While this data is sparse, with an average of fewer than 10 foci reported per study, there are large-scale coordinate databases [e.g. BrainMap (Laird et al. 2005), Neurosynth (Yarkoni et al. 2011)] that index thousands of studies. Hence, CBMA remains the predominant approach for neuroimaging meta-analysis.

To identify brain regions with consistent activation across studies, researchers have developed a variety of CBMA methods, which are either kernel-based or model-based. Kernel-based CBMA methods utilize spatial kernel functions to model the uncertainty around each reported focus. In contrast, model-based CBMA methods employ nuanced statistical models with assumptions about the underlying brain function. Among those kernel-based methods, activation likelihood estimation (ALE, with a Gaussian kernel), multilevel kernel density analysis (MKDA, with a uniform sphere) and signed differential mapping (SDM, with a Gaussian kernel scaled by effect size) are commonly used (Turkeltaub et al. 2002; Wager et al. 2007; Eickhoff et al. 2012; Radua et al. 2012). None of the three methods is based on a formal statistical model, however, all are able to obtain statistical inferences by referencing to a null hypothesis of total random arrangement of the foci (Samartsidis et al. 2017). Voxels with significant *P*-values are considered regions of consistent activation. Multiple testing corrected inferences are made by controlling the family-wise error rate using the null maximum distribution (Westfall and Young 1993) or the false discovery rate (FDR) (Benjamini-Hochberg (BH) procedure, Benjamini and Hochberg 1995). However, kernel-based methods lack interpretability, generally do not allow group comparison, do not model the spatial dependence of activation foci, nor can accommodate study-level covariates to conduct a meta-regression (Samartsidis et al. 2019).

Bayesian model-based methods address these limitations, and are categorized into parametric spatial point process models (Kang et al. 2011; Montagna et al. 2018; Samartsidis et al. 2019) and non-parametric Bayesian models (Yue et al. 2012; Kang et al. 2014). They use explicit generative models for the data with testable assumptions. Although they generally provide advances in interpretability and accuracy over kernel-based methods, they are computationally intensive approaches and generally require parallel computing on GPUs (Samartsidis et al. 2019), and only some approaches can conduct meta-regression to estimate the effect of study-level covariates. Further, it can be more challenging for practitioners to interpret the spatial posterior intensity functions and utilize spatial Bayesian models in practice.

In this work, we propose classical frequentist models that explicitly account for the spatial structure of the distribution of activation foci. Specifically, we develop a spatial model that takes the form of a generalized linear model (GLM), where we make use of a spline parameterization to induce a smooth response and model the entire image jointly; we allow for image-wise study-level regressors and consider different stochastic models to find the most accurate but parsimonious fit. Although Poisson is the classic distribution for describing independent foci counts, we have previously found evidence of over-dispersion (Samartsidis et al. 2020), and thus we further explore a Negative Binomial model, a Clustered Negative Binomial model and a Quasi-Poisson model to allow excess variation in counts data.

Our work builds on the existing methods for CBMA, while introducing key innovations. From the Bayesian work, we adopt the concept of explicit spatial models; from the kernel methods, we incorporate the idea of fixing the degree of spatial smoothness. The contribution of this meta-regression model is both methodological and practical—it provides a generative regression model that estimates a smooth intensity function and can incorporate study-level regressors. Meanwhile, using a crucial memory-saving model factorization, it also offers a computationally efficient alternative to existing Bayesian spatial regression models and provides an accurate estimation of the intensity function. While our method is suitable for any CBMA data, we are particularly motivated by studies of cognition. Cognition encompasses various mental processes, including perception, intelligence, problem solving, social interactions, and can be affected by substance use. We demonstrate this meta-regression framework on previously published meta-analyses of 20 cognitive and psychological tasks, allowing generalized linear hypothesis testing on spatial effect, as well as inference on the effect of study-level covariates.

In the remainder of this work, we present our proposed meta-regression framework, introduce the model factorization and optimization procedures, as well as inferences on meta-regression outcomes via statistical tests in Section 2. Then we explain the experiment settings in Section 3 and explore different variants of stochastic models on the 20 meta-analytic datasets. We describe multiple goodness-of-fit statistics to identify the most accurate model, establish valid FPR control via Monte Carlo simulation under the null hypothesis of spatial homogeneity, followed by a comparison of homogeneity tests with kernel methods in Section 4. Finally, Section 5 summarizes our findings and discusses potential extension of this meta-regression framework in the future.

## 2 Methods

GLMs are described in terms of their stochastic and deterministic components. Our deterministic model features a regression structure with a spatial component utilizing spline parameterization and a study-level covariate component. For the stochastic model, we consider multiple models motivated by CBMA data characteristics. We then propose a model factorization approach to make our methods scalable, before outlining a general inference framework.

### 2.1 Deterministic model

#### 2.1.1 Generic regression structure

Assume there are *N* voxels in each of *M* studies, and then our CBMA data at voxel *j* for study *i* is the voxelwise count of foci *Y_ij_*, written as a *N*-vector $Y_{i}={\left[Y_{i1},Y_{i2},\ldots ,Y_{iN}\right]}^{⊤}$ for study *i*. We generate a spatial design matrix *X* (N×P) with *P* cubic B-spline bases (more details to follow in Section 2.1.2) and construct a study-level covariates matrix *Z* (M×R) using *R* study-level covariates from each of *M* studies. For the CBMA framework, the central object of interest is the voxelwise intensity function for study *i*, which considers both effects of smooth spatial bases and study-level covariates. In this setting, we concisely write the model for study *i* as

(2.1) $$\text{log}(\mu_{i})=\text{log}\;\left[E(Y_{i})\right]=X\beta +(Z_{i}\gamma )1_{N}$$

where *β* (P×1) and *γ* (R×1) are regression coefficients for spatial bases *X* and study-level covariates *Z*, respectively, *Z_i_* is the *i*th row of study-level regressors *Z*, and $1_{N}$ is a *N*-vector of 1’s; the estimated intensity is *μ_ij_* for studies i=1,…,M and voxels j=1,…,N, written as the *N*-vector $\mu_{i}={\left[\mu_{i1},\mu_{i2},\ldots \mu_{iN}\right]}^{⊤}$ for study *i*. This model is identifiable as long as we ensure each covariate variable is mean zero, letting *X* capture the overall mean. The GLM for all voxels in all *M* studies is then

(2.2) $$\text{log}\;\left[E(Y)\right]=(1_{M}⊗X)\beta +(Z⊗1_{N})\gamma$$

where $Y={\left[Y_{1},Y_{2},\ldots ,Y_{M}\right]}^{⊤}$ is a (M×N)-vector, containing voxelwise foci count for all of *M* studies, and ⊗ is the Kronecker product. Note that our GLM has millions of rows (*MN*) and the spatial design matrix has billions of entries (*MN* × *P*). In consideration of implementation complexity and memory requirement, we will propose a simplified reformulation of this GLM in Section 2.3.

#### 2.1.2 Spline parameterization

Previous work on spatial point process modeling of CBMA data has treated each study’s foci as a realization of a doubly-stochastic Poisson process, also known as a Cox process. In some of that work, the log intensity function is parameterized by superimposed Gaussian kernel basis functions (Montagna et al. 2018), while in others, the log intensity is a Gaussian process (Samartsidis et al. 2019). Both the tensor product of cubic B-spline bases and the Gaussian kernel basis functions are suitable for modeling spatial intensity. Their smoothness, stability and ability to provide local support make them ideal spatial bases for CBMA applications. We choose tensor product splines for this work but, in a small evaluation, found that these two approaches have comparable performance; see Appendix S3.1 of the Supplementary Material.

**Fig. 1:**
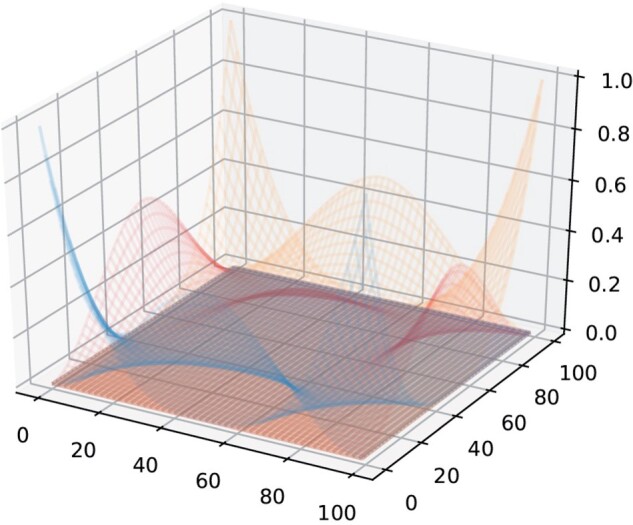
Illustration of a 2D tensor product spline basis.

A 1-dimensional cubic B-spline is a piece-wise polynomial of order 3, where pre-specified knots determine the parameterization of basis functions where the polynomial sections join. For our 3D lattice, assume there are *v_x_* voxels along the x direction, the coefficients of *v_x_* voxels evaluated at each of *n_x_* B-spline bases construct a coefficient matrix *C_x_* (size $v_{x}\times n_{x}$). Similarly, there exist another two coefficient matrices *C_y_* and *C_z_* (size $v_{y}\times n_{y}$ and $v_{z}\times n_{z}$) along y and z direction. The whole coefficient matrix *C* of 3-dimensional B-spline bases is constructed by taking the tensor product of the three coefficient matrices (see Fig. 1 for a 2D illustration),

(2.3) $$C=C_{x}⊗C_{y}⊗C_{z}$$

The matrix of *C* is $\left(v_{x}v_{y}v_{z})\times (n_{x}n_{y}n_{z}\right)$, and is the basis for the entire 3D volume, while the analysis is based on a brain mask of *N* voxels. The design matrix *X* is obtained from *C* after a three-step process: First, rows corresponding to voxels outside the brain mask are removed; then, columns are removed if they correspond to weakly supported B-spline bases (a B-spline basis is regarded as “weakly supported” if its maximum value of coefficients evaluated at each voxel is below 0.1). Finally, the rows are re-normalized (sum to 1) to preserve the “partition of unity” property of B-spline bases.

We define our cubic B-spline bases with equally spaced knots in *x*, *y*, and *z* dimensions, and thus we parameterize the level of spatial smoothness by the knot spacing. Larger knots spacing, smaller basis, and greater smoothness; conversely, closer knots, larger basis, and greater ability to represent fine details. Conceptually, more flexible parameterizations would allow arbitrary knots locations, but with the consideration of minimizing computational complexity, we fix the design matrix *X* based on pre-specified knots spacing and locations. We also provide practical recommendations on parameter selection for knot configuration in Section S3.2 in the Supplementary Material. While other spline applications use a dense array of knots and then control smoothness with a roughness penalty, the computational and memory requirements of our spatial model demand that we judiciously select the coarsest spline spacing consistent with our application.

### 2.2 Stochastic model

Different stochastic assumptions on the CBMA foci data determine the form of statistical likelihood we use. We consider a set of four stochastic models for the distribution of foci counts at the voxel level. All of our models take the form of GLMs, where inhomogeneous intensity at each voxel is captured by the spline bases and any study-level covariate (as per Equation (2.2)). We fit our model either by maximizing log-likelihood function iteratively via L-BFGS (Limited-memory Broyden–Fletcher–Goldfarb–Shanno) algorithm (Shanno 1970) for likelihood-based models or iteratively re-weighted least squares (IRLS) for Quasi-likelihood models. We now elaborate each of these models in turn and discuss their strengths and limitations.

#### 2.2.1 Poisson model

In practice, the count of foci *Y_ij_* (for studies i=1,…,M, voxels j=1,…,N) is only ever 0 or 1, which strictly indicates a Binomial model. However, inspired by previous success with the Poisson point process, and the accuracy of the Poisson approximation for low-rate Binomial data (Eisenberg et al. 1966), we consider a Poisson model.

If foci arise from a realization of a (continuous) inhomogeneous Poisson process, the (discrete) voxel-wise counts will be independently distributed as Poisson random variables, with a rate equal to the integral of the (true, unobserved, continuous) intensity function over each voxel. As the sum of multiple independent Poisson random variables is also Poisson, this also gives rise to a practical consequence: it is equivalent to either model the set of *M* study-level counts or the summed counts at each voxel. Following the deterministic structure outlined in Equation (2.1), the intensity for voxel *j* in study *i* is

(2.4) $$\begin{array}{cc} E[Y_{ij}] & =\mu_{ij}\\ \;\text{log}(\mu_{ij}) & =\eta_{ij}=x_{j}^{⊤}\beta +Z_{i}\gamma \end{array}$$

where $Y_{ij}∼\text{Poisson}(\mu_{ij}),\;x_{j}^{⊤}$ is the *j*th row of spatial design matrix X(N×P), and *β* is the regression coefficient of spline bases. The data vector *Y* has a length-(*MN*), which is impractical to represent explicitly. Under the assumption of independence of counts across studies, the likelihood function is exactly the same if we model the voxel-wise total foci count over studies instead (more details to follow in S1.1 in the Supplementary Material), which gives rise to the modified Poisson model on summed data at voxel *j* over all studies, $Y_{\cdot ,j}=\sum_{i=1}^{M}Y_{ij}$,

(2.5) $$\begin{array}{cc} E[Y_{\cdot ,j}]=\mu_{\cdot ,j}, & \\ \mu_{\cdot ,j}=\sum_{i=1}^{M}\mu_{ij}=\sum_{i=1}^{M}\;\text{exp}\;\left(x_{j}^{⊤}\beta +Z_{i}\gamma \right)=\text{exp}(x_{j}^{⊤}\beta )\left(\sum_{i=1}^{M}\;\text{exp}\;(Z_{i}\gamma )\right) & \end{array}$$

where $\mu_{\cdot ,j}=\sum_{i=1}^{M}\mu_{ij}$ is the expected sum of intensity at voxel *j* over studies. Under this formulation, the likelihood to be optimized is,

(2.6) $$l(\theta )=l(\beta ,\gamma )=\sum_{j=1}^{N}\left[Y_{\cdot ,j}\;\text{log}(\mu_{\cdot ,j})-\mu_{\cdot ,j}-\text{log}(Y_{\cdot ,j}!)\right]$$

#### 2.2.2 Negative Binomial model

While Poisson model is widely used in the regression of count data, it is recognized that counts often display over-dispersion (the variance of the response variable substantially exceeds the mean). Imposing a Poisson model based on the unrealistic assumption (variance equals mean) may underestimate the standard error, and lead to biased estimation of the regression coefficients. While Barndorff-Nielsen and Yeo (1969) proposed a formal definition of spatial Negative Binomial model, it involves Gaussian processes and complexities we sought to avoid. Hence, here we do not propose a formal point process model, but rather simply assert that the count data at each voxel follows a Negative binomial (NB) distribution independently, thus allowing for anticipated excess variance relative to Poisson (Lawless 1987).

Our NB model uses a single parameter *α* shared over all studies and all voxels to index variance in excess of the Poisson model. For each study *i* and voxel *j*, let *λ_ij_* follow a Gamma distribution with mean *μ_ij_* and variance $\alpha \mu_{ij}^{2}$; then conditioned on *λ_ij_*, let *Y_ij_* be Poisson with mean *λ_ij_*. It can be shown that the marginal distribution of *Y_ij_* follows a NB distribution with probability mass function,

(2.7) $$P(Y_{ij}=y_{ij})=\frac{\Gamma (y_{ij}+\alpha^{-1})}{\Gamma (y_{ij}+1)\Gamma (\alpha^{-1})}{\left(\frac{1}{1+\alpha \mu_{ij}}\right)}^{\alpha^{-1}}{\left(\frac{\alpha \mu_{ij}}{1+\alpha \mu_{ij}}\right)}^{y_{ij}}.$$

In terms of the success count and probability parameterization, NB(r,p), we have $Y_{ij}∼\text{NB}(\alpha^{-1},\frac{\mu_{ij}}{\alpha^{-1}+\mu_{ij}})$, with mean $E(Y_{ij})=\mu_{ij}$ and variance $V(Y_{ij})=\mu_{ij}+\alpha \mu_{ij}^{2}$. Details on the derivation of the probability density function of the NB model can be found in S1.2 of the Supplementary Material. When α>0, we observe Poisson-excess variance of $\alpha \mu_{ij}^{2}$; or analogous to the coefficient of variation, the coefficient of excess variation is $\sqrt{\alpha \mu_{ij}^{2}}/\mu_{ij}=\sqrt{\alpha }$, which can be interpreted roughly as the relative excess standard deviation relative to a Poisson model.

Again, the data vector is impractical to represent explicitly, but unlike Poisson, the sum of multiple independent NB random variables doesn’t follow an NB distribution. Thus, we propose a moment matching approach to approximate the mean (first moment) and variance (second moment) of this convolution of NB distributions, which significantly facilitates the simplification of the log-likelihood function. Matching the first two moments, the approximate NB distribution of the total count of foci over all studies at voxel *j* is given by $Y_{\cdot ,j}=\sum_{i=1}^{M}Y_{ij}∼\text{NB}(r\prime_{j},p\prime_{j})$, where

$$r\prime_{j}=\frac{\mu_{\cdot ,j}^{2}}{\alpha \sum_{i=1}^{M}\mu_{ij}^{2}},\;\;p\prime_{j}=\frac{\sum_{i=1}^{M}\mu_{ij}^{2}}{\alpha^{-1}\mu_{\cdot ,j}+\sum_{i=1}^{M}\mu_{ij}^{2}}$$

with corresponding excess variance

$$\alpha \prime =\alpha \frac{\sum_{i=1}^{M}\mu_{ij}^{2}}{\mu_{\cdot ,j}^{2}},$$

which gives rise to the simplified NB log-likelihood function,

(2.8) $$\begin{array}{c} l(\theta )\approx l(\beta ,\alpha \prime )\\ \;=\sum_{j=1}^{N}\left[\text{log}\;\Gamma (Y_{\cdot ,j}+r\prime_{j})-\text{log}\;\Gamma (Y_{\cdot ,j}+1)-\text{log}\;\Gamma (r\prime_{j})+r\prime_{j}\;\text{log}(1-p\prime_{j})+Y_{\cdot ,j}\;\text{log}\;p\prime_{j}\right] \end{array}$$

Details on the derivations of the moment matching approach can be found in S1.3 in the Supplementary Material. We have also included a simulation in Section S1.4 in the Supplementary Material, which demonstrates the accuracy of this method in approximating the sum of NB distributed variates. Our findings indicate a negligible bias (0.3098%) in the standard error estimate for the mean estimate using the moment matching approach. Furthermore, the maximum likelihood estimates (MLEs) for both methods are remarkably close to their true values.

#### 2.2.3 Clustered Negative Binomial model

While the NB model can be regarded as a kind of “random effects” Poisson model, as developed above, the latent Gamma random variable introduces independent variation at each voxel. Instead, we could assert that the random (Gamma-distributed) effects are not independent voxel-wise effects, but rather latent characteristics of each study, representing a shared effect over the entire brain for a given study. This is, in fact, the approach used by a Bayesian CBMA method (Samartsidis et al. 2019), and in a non-imaging setting, a Poisson-Gamma model for two-stage cluster sampling (Geoffroy and Weerakkody 2001). Therefore, we now consider a third GLM, where at the first stage, we assume each individual study *i* is sampled with a global latent value *λ_i_* from a Gamma distribution with mean 1 and variance *α*, which accommodates excess variance by the dispersion parameter *α* ($\lambda_{i}∼\mathrm{Gamma}(\alpha^{-1},\alpha^{-1})$). At the second stage, conditioned on the global variable *λ_i_*, *Y_ij_* are drawn from a Poisson distribution with mean $\lambda_{i}\mu_{ij}$ ($Y_{ij}|\lambda_{i}∼\text{Poisson}(\lambda_{i}\mu_{ij})$), where *μ_ij_* is the expected intensity parameterized by spatial regression parameter *β* and covariates regression parameter *γ*. The marginal distribution of *Y_ij_* also follows an NB distribution,

(2.9) $$P(Y_{ij}=y_{ij})=\frac{\Gamma (y_{ij}+\alpha^{-1})}{\Gamma (y_{ij}+1)\Gamma (\alpha^{-1})}{\left(\frac{\alpha^{-1}}{\mu_{ij}+\alpha^{-1}}\right)}^{\alpha^{-1}}{\left(\frac{\mu_{ij}}{\mu_{ij}+\alpha^{-1}}\right)}^{y_{ij}}$$

where $Y_{ij}∼NB(\alpha^{-1},\frac{\mu_{ij}}{\alpha^{-1}+\mu_{ij}})$ with mean $E(Y_{ij})=\mu_{ij}$ and variance $V(Y_{ij})=\mu_{ij}+\alpha \mu_{ij}^{2}$. Details on the derivation of the probability density function of the clustered NB model can be found in S1.5 in the Supplementary Material. This two-stage hierarchical Clustered NB model also introduces a covariance structure between foci within a study, which is determined by the expected intensity of the observations as well as the dispersion parameter *α* (see S1.6 in the Supplementary Material). The covariance for studies *i* and i′, and distinct voxel *j* and j′ is,

(2.10) $$\{\begin{array}{c} C(Y_{ij},Y_{i\prime ,j\prime })=\alpha \mu_{ij}\mu_{ij\prime },\;\text{if}\;i=i\prime \\ C(Y_{ij},Y_{i\prime ,j\prime })=0,\;\text{if}\;i\neq i\prime \end{array}$$

The log-likelihood is the sum of terms over independent studies,

(2.11) $$\begin{array}{c} l(\beta ,\alpha ,\gamma )=\sum_{i=1}^{M}\;\text{log}\;[f(Y_{i1},Y_{i2},\ldots ,Y_{iN})]\\ =M\alpha^{-1}\;\text{log}(\alpha^{-1})-M\;\text{log}\;\Gamma (\alpha^{-1})+\sum_{i=1}^{M}\;\text{log}\;\Gamma (Y_{i,\cdot }+\alpha^{-1})\\ -\sum_{i=1}^{M}\sum_{j=1}^{N}\;\text{log}\;Y_{ij}!-\sum_{i=1}^{M}\left(Y_{i,\cdot }+\alpha^{-1})\;\text{log}(\mu_{i,\cdot }+\alpha^{-1}\right)+\sum_{i=1}^{M}\sum_{j=1}^{N}Y_{ij}\;\text{log}\;\mu_{ij} \end{array}$$

where $Y_{i,\cdot }=\sum_{j=1}^{N}Y_{ij}$ is the sum of foci within study *i*. One limitation of this model, though, is that it doesn’t admit a factorization and depends on the length-(MN) data vector (see S1.6 in Supplementary Material).

While the intra-study dependence is well-motivated, the Clustered NB model depends on the strong assumption that excess variance is captured by the global dispersion *λ_i_*. If there is voxel-wise independent excess variance, the previous NB model will be preferred; we assess this issue below with real data evaluations.

#### 2.2.4 Quasi-Poisson model

As an alternative to the NB model, Quasi-Poisson model also allows for over-dispersed count data, and is a straightforward elaboration of the GLM. Instead of specifying a specific probability distribution for count data, the Quasi-Poisson model only requires a mean model and a variance function, $V(Y_{ij})=\theta \mu_{ij}$ (with θ≥1). While the variance-mean relationship is linear for the Quasi-Poisson model, it is quadratic in the NB model. This results in small foci counts being weighted more and can have greater adjustment effect in the Quasi-Poisson model, which theoretically might be ideal for our scenario where most brain regions have zero or low foci counts (Ver Hoef and Boveng 2007).

Quasi-Poisson model can be framed as a GLM, with the mean and variance for voxel *j* in study *i* given by,

(2.12) $$\begin{array}{cc} E[Y_{ij}] & =\mu_{ij}\\ \text{Var}(Y_{ij}) & =\theta \mu_{ij}. \end{array}$$

Without a likelihood function, we instead use ILRS algorithm, with the (k+1)th iteration given by,

(2.13) $$\begin{array}{cc} {\hat{\beta }}^{\left[k+1\right]} & =\beta^{\left[k\right]}+{\left(X^{*⊤}W^{\left[k\right]}X^{*}\right)}^{-1}X^{*⊤}(Y-\mu^{\left[k\right]})\\ {\hat{\gamma }}^{\left[k+1\right]} & ={\hat{\gamma }}^{\left[k\right]}+{\left(Z^{*⊤}W^{\left[k\right]}Z^{*}\right)}^{-1}Z^{*⊤}(Y-\mu^{\left[k\right]}) \end{array}$$

where $W=\text{diag}(\frac{\mu_{11}}{\theta },\ldots ,\frac{\mu_{1N}}{\theta },\ldots ,\frac{\mu_{M1}}{\theta },\ldots ,\frac{\mu_{MN}}{\theta })$, and $X^{*}=1_{M}⊗X,\;Z^{*}=1_{N}⊗Z$. This model can be simplified as well, though we again defer that to Section 2.3.

### 2.3 Model factorization

Having derived the explicit log-likelihood functions for meta-regression with three stochastic likelihood-based models, as well as the updating equation for a quasi-likelihood based model, we now consider model factorization to replace the full (*MN*)-vector of foci counts by sufficient statistics. Following the generic formulation of GLM proposed in Section 2.1.1,

(2.14) $$\eta_{ij}=\text{log}(\mu_{ij})=\sum_{k=1}^{P}X_{jk}\beta_{k}+\sum_{s=1}^{R}Z_{is}\gamma_{s}.$$

*η_ij_* is the estimated linear response from GLM, specific to each voxel *j* in each individual study *i*. In our neuroimaging application, there are always at least 220, 000 voxels (*N*), hundreds or thousands of studies *M*, and ≈500 or more basis elements (*P* = 456 for 20*mm* knots spacing), giving rise to millions of rows (*MN*) and billions of entries (MN×(P+R)) in a GLM formulation. Thus, we propose a reformulation of this model into a series of sufficient statistics that are never larger than *M* or *N* in dimension. First, note that the localized spatial effect *μ^X^* and global effect of study-level covariates $\mu_{i}^{Z}$ for study *i* factorize *μ_ij_* as

(2.15) $$\mu_{ij}=\text{exp}\;\left(\sum_{k=1}^{P}X_{jk}\beta_{k}+\sum_{s=1}^{R}Z_{is}\gamma_{s}\right)=\text{exp}\;\left(\sum_{k=1}^{P}X_{jk}\beta_{k}\right)\;\text{exp}\;\left(\sum_{s=1}^{R}Z_{is}\gamma_{s}\right)=\mu_{j}^{X}\mu_{i}^{Z}$$

To further simplify the log-likelihood function, we also use the fact that $Y_{ij}\leq 1$ (either 0 or 1), as there will never be more than one foci at the same location in a given study. Define the following notation:

- Let N-vector $\mu^{X}=\text{exp}(X\beta )$ be the vector of spatial effects;
- let *M*-vector $\mu^{Z}=\text{exp}(Z\gamma )$ be the vector of global study-level covariates effects;
- as already defined, $Y_{\cdot ,j}=\sum_{i=1}^{M}Y_{ij}$ is sum of foci counts at voxel *j* across all studies, and define the *N*-vector $Y_{\cdot ,}={\left[Y_{\cdot ,1},\ldots ,Y_{\cdot ,N}\right]}^{⊤}$;
- and let $Y_{i,\cdot }=\sum_{j=1}^{N}Y_{ij}$ be the sum of foci counts for study *i* across all voxels, and define the *M*-vector $Y_{,\cdot }={\left[Y_{1,\cdot },\ldots ,Y_{M,\cdot }\right]}^{⊤}$.

The simplified factorization of total log-likelihood functions or IRLS updating equation are specific to each stochastic model. Full details are provided in S2 in the Supplementary Material; in summary:

- Poisson model:
  (2.16) $$l(\beta ,\alpha )=Y_{\cdot ,}^{⊤}\;\text{log}(\mu^{X})+Y_{,\cdot }^{⊤}\;\text{log}(\mu^{Z})-\left[1^{⊤}\mu^{X}\right]\left[1^{⊤}\mu^{Z}\right],$$
- NB model: As described in Section 2.2.2, we approximate a sum of independent NB variables again as a NB:
  (2.17) $$Y_{\cdot ,j}=\sum_{i=1}^{M}Y_{ij}∼\text{NB}(r\prime_{j},p\prime_{j})=\text{NB}\left(\frac{{\left(\mu_{j}^{X}\right)}^{2}{\left[1^{⊤}\mu^{Z}\right]}^{2}}{\alpha \prime \sum_{i=1}^{M}{\left(\mu_{j}^{X}\mu_{i}^{Z}\right)}^{2}},\frac{\sum_{i=1}^{M}{\left(\mu_{j}^{X}\mu_{i}^{Z}\right)}^{2}}{{\left(\alpha \prime \right)}^{-1}\mu_{j}^{X}{\left[1^{⊤}\mu^{Z}\right]}^{⊤}+\sum_{i=1}^{M}{\left(\mu_{j}^{X}\mu_{i}^{Z}\right)}^{2}}\right)$$
  with dispersion parameter $\alpha \prime =\frac{\alpha \sum_{i=1}^{M}{\left(\mu_{j}^{X}\mu_{i}^{Z}\right)}^{2}}{{\left(\mu_{j}^{X}\right)}^{2}{\left[1^{⊤}\mu^{Z}\right]}^{2}}$. The log-likelihood function is given by,
  (2.18) $$\begin{array}{c} l(\alpha \prime ,\beta ,\gamma )=\sum_{j=1}^{N}[\;\text{log}\;\Gamma (Y_{\cdot ,j}+r\prime_{j})-\text{log}\;\Gamma (Y_{\cdot ,j}+1)-\text{log}\;\Gamma (r\prime_{j})\\ \;\;\;\;+r\prime_{j}\;\text{log}(1-p\prime_{j})+Y_{\cdot ,j}\;\text{log}\;p\prime_{j}], \end{array}$$
  Clustered NB model:
  (2.19) $$\begin{array}{cc} l(\alpha ,\beta ,\gamma ) & =M\alpha^{-1}\;\text{log}(\alpha^{-1})-M\;\text{log}\;\Gamma (\alpha^{-1})+\sum_{i=1}^{M}\;\text{log}\;\Gamma (Y_{i,\cdot }+\alpha^{-1})\\ & -\sum_{i=1}^{M}\left(Y_{i,\cdot }+\alpha^{-1})\;\text{log}(\alpha^{-1}+\mu_{i,\cdot })+Y_{\cdot ,}^{⊤}\;\text{log}(\mu^{X})+Y_{,\cdot }^{⊤}\;\text{log}(\mu^{Z}\right) \end{array}$$
  where dispersion parameter *α* measures the excess variance across all studies and all voxels,
- Quasi-Poisson model:
  (2.20) $$\begin{array}{cc} {\hat{\beta }}^{\left[j+1\right]} & =\beta^{\left[j\right]}+{\left(X^{⊤}W^{\left[j\right]}X\right)}^{-1}X^{⊤}(Y_{\cdot ,}-{\left(\mu^{X}\right)}^{\left[j\right]})\\ {\hat{\gamma }}^{\left[j+1\right]} & ={\hat{\gamma }}^{\left[j\right]}+{\left(Z^{⊤}V^{\left[j\right]}Z\right)}^{-1}Z^{⊤}(Y_{,\cdot }-{\left(\mu^{Z}\right)}^{\left[j\right]}) \end{array}$$
  where $W=\text{diag}(\frac{\mu_{1}^{X}}{\theta },\ldots ,\frac{\mu_{N}^{X}}{\theta })$ and $V=\text{diag}(\frac{\mu_{1}^{Z}}{\theta },\frac{\mu_{2}^{Z}}{\theta },\ldots ,\frac{\mu_{M}^{Z}}{\theta })$.

### 2.4 Model optimization

For likelihood-based models (Poisson, NB, and clustered NB model; Section 2.2.1–Section 2.2.3) without study-level covariates, we employ Fisher scoring for the iterative optimization of parameters in GLMs. Fisher scoring replaces the gradient and Hessian of Newton’s method with the score and observed Fisher’s information, respectively (Longford 1987). Writing *θ* for all parameters, the updating equation at the (k+1)th iteration is,

(2.21) $$\theta^{\left[k+1\right]}=\theta^{\left[k\right]}+I{\left(\theta^{\left[k]\right)}\right)}^{-1}\frac{\partial }{\partial \theta^{\left[k\right]}}l(\theta^{\left[k]\right)})$$

where the observed Fisher information is $I(\theta^{\left[k\right]})=E{\left[-\frac{\partial^{2}l(\theta )}{\partial \theta \partial \theta^{⊤}}\right]}_{\theta =\theta^{\left[k\right]}}$.

For the Poisson model, θ=[β,γ], the Fisher information is given by,

(2.22) $$I(\theta )=I(\beta ,\gamma )=\left[\begin{array}{cc} -\frac{\partial^{2}l}{\partial \beta \partial \beta^{⊤}} & -\frac{\partial^{2}l}{\partial \beta \partial \gamma^{⊤}}\\ -\frac{\partial^{2}l}{\partial \gamma \partial \beta^{⊤}} & -\frac{\partial^{2}l}{\partial \gamma \partial \gamma^{⊤}} \end{array}\right]$$

with negative Hessian matrix of *β*, ${\left(-\frac{\partial^{2}l}{\partial \beta \partial \beta^{⊤}}\right)}_{P\times P}=X^{⊤}\text{diag}(\mu^{X})X$; the negative cross term ${\left(-\frac{\partial^{2}l}{\partial \beta \partial \gamma^{⊤}}\right)}_{P\times R}={\left(-\frac{\partial^{2}l}{\partial \gamma \partial \beta^{⊤}}\right)}_{R\times P}^{⊤}=[X^{⊤}\mu^{X}][{\left(\mu^{Z}\right)}^{⊤}Z]$; and negative Hessian matrix of *γ*, $\left(-\frac{\partial^{2}l}{\partial \gamma \partial \gamma^{⊤}}\right)=Z^{⊤}\text{diag}(\mu^{Z})Z$.

Likelihood-based models with study-level covariates lead to more complicated derivations of updating equations via Fisher scoring. Instead, we use a more efficient quasi-Newton algorithm (the L-BFGS algorithm, Shanno 1970), which minimizes smooth, nonlinear functions without directly computing the Hessian matrix. Instead, it estimates the observed Fisher Information with gradient evaluations, significantly reducing both memory requirements and computational complexity. It is particularly well-suited for optimization problems characterized by a large number of variables, where computing the full Hessian matrix would be computationally expensive or even infeasible due to memory constraints.

Lastly, for Quasi-likelihood models (e.g. Quasi-Poisson model, see Section 2.2.4) where exact likelihood functions are computationally infeasible, optimizing the regression coefficients using Fisher scoring becomes impractical. Instead, we employ the Iteratively Reweighted Least Squares (IRLS) method to iteratively find the optimal regression coefficients. For the updating equations, please refer to Section S2.4 in the Supplementary Material.

### 2.5 Statistical inference

#### 2.5.1 Global test of model fitness

Among the proposed stochastic models in Section 2.2, the Poisson, NB and clustered NB model are likelihood-based, while Quasi-Poisson model is Quasi-likelihood based (its exact likelihood is computationally infeasible). To compare the goodness of fit from a global perspective, we will utilize likelihood-based comparison criteria (e.g. LRT and Akaike information criterion (AIC)) with likelihood-based models, as well as other global model fitness criteria across all stochastic models within this meta-regression framework.

##### Likelihood-based model selection criteria

LRT uses the difference in log-likelihoods to test the null hypothesis that the true model is the smaller nested model. Since the Poisson model is nested in both the NB model and the clustered NB model with a dispersion parameter *α* = 0, for the null hypothesis *H*_0_: dispersion parameter *α* = 0, the likelihood-ratio test statistic is given by,

$$\lambda_{LR}=-2\left[l({\hat{\theta }}_{0})-l(\hat{\theta })\right]$$

where $l(\hat{\theta })=l(\hat{\alpha },\hat{\beta },\hat{\gamma })$ is the maximum log-likelihood of the NB model or clustered NB model without any constraint on parameters, and $l({\hat{\theta }}_{0})=l(\hat{\alpha }=0,\hat{\beta },\hat{\gamma })$ is the maximum log-likelihood of the NB model or clustered NB model with the dispersion parameter *α* constrained at 0 (ie Poisson model). The test statistic is Chi-square distributed with 1 degree of freedom.

AIC is an alternative to LRT which also addresses the trade-off between the goodness of fit and the simplicity of the model, and it addresses the overfitting problem by penalizing the number of parameters in the model. To measure the goodness of fit of a model *M* on dataset *D*,

(2.23) $$\mathrm{AIC}=2k-2l(\hat{\theta })$$

where $l(\hat{\theta })$ is the maximized log-likelihood function of the model *M*, *k* is the number of parameters in model *M*. The model with the smaller AIC is believed to be a better fit to the dataset.

##### Bias and variance of estimation

For the purpose of selecting the best model in terms of goodness of fit across a variety of datasets, we extend the model comparisons to include all stochastic models proposed in Section 2.2, including the Quasi-Poisson model. As the central outcome of this meta-regression framework is voxel-wise intensity estimation for each study, with the effect of study-level covariates being considered, it’s natural to utilize bias and variance of intensity estimation as new criteria stated below,

- Relative bias of the estimated total sum of intensity (per study), comparing with the averaged sum of foci counts (per study) across multiple datasets;
- Relative bias of standard deviation (SD) in each of *x*, *y*, *z* dimension, compared with the actual standard deviation in foci count (per study) across multiple datasets;
- Relative bias of voxel-wise variance between the actual foci count (per study) and the intensity estimation (per study).

Here, relative bias is evaluated instead of absolute bias, especially when applied to a variety of datasets with diverse foci counts.

#### 2.5.2 Localized inference with Wald tests on $\mu_{ij}^{X}$ and $\eta_{ij}^{X}$

While our model is parameterized by *P* basis elements, users want to make inference at each of the *N* voxels. Hence, we provide localized inference on estimated spatial intensity $\mu_{ij}^{X}$ (or $\eta_{ij}^{X}=\text{log}(\mu_{ij}^{X})$) and the regression coefficient of study-level covariates (*γ*) via Wald tests.

##### Test of spatial homogeneity

In the CBMA context, the most basic inference is a test of homogeneity to identify regions where more foci arise than would be expected if there were no spatial structure. Precisely, we use the null hypothesis on voxelwise intensity estimation or estimated linear response, $H_{0}:\mu_{ij}^{X}=\mu_{0}=\sum_{i=1}^{M}\sum_{j=1}^{N}Y_{ij}/(MN)$ or $\eta_{ij}^{X}=\eta_{0}=\text{log}(\mu_{0})$ at voxel *j*, for study *i*. The standard error for *β* can be asymptotically estimated from the inverse of the observed Fisher Information matrix, which gives rise to the standard error for the linear response $\eta_{ij}^{X}$, and thus the standard error for $\mu_{ij}^{X}$ is obtained via the delta method (see Section S2.5 in the Supplementary Material for details). It allows inference via Wald tests by examining voxelwise intensity estimation against the null hypothesis of homogeneity over space. The signed Wald statistic for $\mu_{ij}^{X}$ or $\eta_{ij}^{X}$ takes the form:

(2.24) $$Z_{\mu^{X}}=\frac{\mu_{ij}^{X}-\mu_{0}}{\text{SE}(\mu_{ij}^{X})},\;\;Z_{\eta^{X}}=\frac{\eta_{ij}^{X}-\eta_{0}}{\text{SE}(\eta_{ij}^{X})}$$

where $SE(\mu_{ij}^{X})$ is the standard error of the estimated spatial intensity $\mu_{ij}^{X}$, and $SE(\eta_{ij}^{X})$ is the standard error of the estimated linear response $\eta_{ij}^{X}$, and the statistics are asymptotically Gaussian. Finally, we can create *P*-value maps that are thresholded to control the false discovery rate (FDR) at 5% (Benjamini and Hochberg 1995).

#### 2.5.3 Inference on study-level covariates

For the regression coefficient *γ* (s×1) of study-level covariates, we consider general linear hypothesis (GLH) tests through a contrast matrix $C_{\gamma }$ (*m* × *s*). Under the null hypothesis,

(2.25) $$H_{0}:C_{\gamma }\gamma =0_{m\times 1}$$

The test statistic follows a $\chi^{2}$ distribution with *m* degree of freedom asymptotically,

(2.26) $${\left(C_{\gamma }\hat{\gamma }\right)}^{T}{\left(C_{\gamma }\text{Cov}(\hat{\gamma })C_{\gamma }^{T}\right)}^{-1}(C_{\gamma }\hat{\gamma })\overset{D}{\to }\chi_{m}^{2}$$

and in the case of a single contrast (*m* = 1), a signed Z test can be computed. Details of GLH on study-level covariates can be found in S4.1 in the Supplementary Material.

## 3 Experiments

### 3.1 Simulation settings

The statistical analyses of model estimation with CBMA data are conducted at the voxel level: voxelwise test statistics are evaluated to examine the significance of the experimental effect. Therefore, before investigating model fitness, we evaluate our models’ false positive rates (FPR) under null settings. Due to the computationally intensive nature of these evaluations, we only evaluated the two models that showed promise in other evaluations, Poisson and NB. Under the null hypothesis of spatial homogeneity, we use Monte Carlo (MC) simulation to establish the validity of FPR control for the test of spatial intensity (*μ^X^*). Specifically, we will explore meta-regression with either the Poisson or NB model, with or without study-level covariates. To ensure the validity of FPR control is applicable to all CBMA data, the sampling mechanism is either model-based or empirical, with simulated foci count always analogous to the foci count within a real dataset. Specifically, in model-based sampling, the data generating mechanism matches the regression model, with the number of studies and average foci per study identical to the original dataset; while in empirical sampling, real data foci locations are randomly shuffled to guarantee the spatial homogeneity of the foci distribution.

### 3.2 Applications to 20 meta-analytic datasets

Cognition concerns psychological and cognitive processes that focus on learning people’s perception, interpretation and response to information and stimuli. It refers to both conscious procedure and unconscious, automatic mechanisms in the brain that occur as a response to stimuli, and is highly variable across individuals (Gallagher et al. 2019). Cognition has been studied intensively to identify brain regions involved in cognition tasks, conducted in an MRI scanner. Here we use 20 previously published meta-analytic datasets for the purpose of evaluating the accuracy and sensitivity of this meta-regression framework, as well as analyzing the goodness of fit of stochastic models with respect to different CBMA datasets. These datasets involve multiple aspects of cognition research, as listed in Table 1.

**Table 1:** Number of contrasts and foci counts of 20 meta-analytic datasets.

Dataset	Number of contrasts	Total count of foci	Max foci count	Average foci count
1. Social processing	599	4934	47	8.24
2. PTSD	22	154	26	7.00
3. Substance use	89	657	110	7.38
4. Dementia	28	1194	548	42.64
5. Cue reactivity	275	3197	58	11.63
6. Emotion regulation	338	3543	87	10.48
7. Decision making	145	1225	49	8.45
8. Reward	850	6791	59	7.99
9. Sleep deprivation	44	454	59	10.32
10. Naturalistic	122	1220	59	10.00
11. Problem solving	282	3043	44	10.79
12. Emotion	1738	22038	203	12.68
13. Cannabis use	81	314	16	3.88
14. Nicotine use	13	77	23	5.92
15. Frontal pole CBP	795	9525	57	11.98
16. Face perception	385	2920	50	7.58
17. Nicotine administration	75	349	24	4.65
18. Executive function	243	2629	54	10.82
19. Finger tapping	76	696	27	9.16
20. n-Back	29	640	69	22.07

**Fig. 2:**
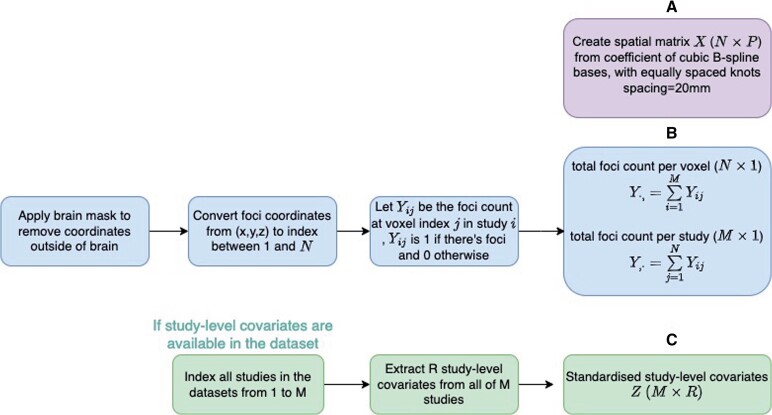
Preprocessing pipeline of meta-analytic datasets before fitting coordinate-based meta-regression (CBMR) framework. Note that panel A and B are applicable to all datasets, which generate a spatial design matrix *X*, total foci count per voxel $Y_{\cdot ,}(N\times 1)$ and total foci count per study $Y_{,\cdot }(M\times 1)$. Panel C is only needed if the effect of study-level covariates is considered, as covariates matrix *Z* (M×R).

The preprocessing steps are summarized in Fig. 2. The discrete sampling space of our analysis is the 2*mm* ^3^ MNI (Montreal Neurogical Institute) atlas (Collins et al. 1994), with dimensions 91×109×91, and *N* = 228, 483 brain voxels. We first apply this brain mask to remove foci outside the brain and remove any multiple-foci (while original data peaks are always distinct, a foci count in excess of 1 can occur when Talairach coordinates are rounded to the MNI 2mm grid). We then extract all the sufficient statistics after model factorization in Section 2.3, including the spatial design matrix *X* (N×P) generated from B-spline bases, total foci count per voxel $Y_{\cdot ,}(N\times 1)$ and total foci count per study $Y_{,\cdot }(M\times 1)$ and study-level covariates Z(M×R) if considered.

## 4 Results

### 4.1 Simulation results

For each of the 20 meta-analytic datasets, we simulate foci distribution under a null hypothesis of spatial homogeneity, estimate spatial intensity and investigate the distribution of voxel-wise *P*-values for the eight different scenarios: fitting Poisson or NB model, using a model-based or empirical (random shuffling) data sampling mechanism, and including or omitting study-level covariates. For all settings, we use a B-spline knot spacing of 20*mm* in *x*, *y*, *z* direction, producing *P* = 456 basis elements. The computation of test statistics depends on the covariance of regression coefficients, which is approximated by the inverse of the Fisher Information matrix of optimized parameters at maximized log-likelihood (see Section 2.4). Empirically, we sometimes found the *P*-values are underestimated, particularly below the threshold of $10^{-3}$, which we believe has two causes. Firstly, the inference based on the inverse Fisher Information (FI) matrix is only asymptotic, and hence under- or over-coverage could be obtained for any finite number of studies *N*. Secondly, small meta-analyses with some regions having essentially no foci drive some of the *β* coefficients to negative infinity, producing an estimated rate of zero, which in turn produces an ill-conditioned and singular FI matrix. (In our experiments, we observed that datasets with a total foci count of at least 1000 generally avoided these singularity problems and produced accurate standard errors for NB model, however, this criterion also depends on the chosen spline knot spacings (we also provide a practical guideline of choosing appropriate knot spacings based on the total foci counts in Section S4.1 in the Supplementary Material). We tried various different approaches to regularize and make the FI matrix invertible but these often deflated the computed sample variances, inflating significance, and hence are not part of the proposed method.

**Fig. 3:**
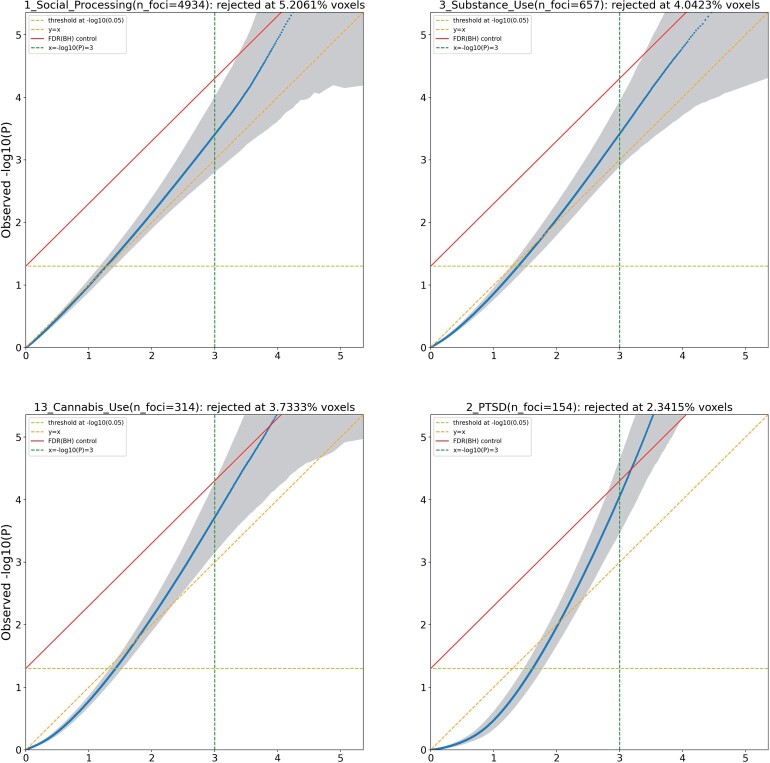
P–*P* plot of null *P*-values, $-{\;\text{log}\;}_{10}$ scale, showing four representative meta-analytic datasets (Social Processing, Substance Use, Cannabis Use and PTSD datasets), estimated by CBMR with NB model without study-level covariates, with null data generated with a model-based approach. CBMR’s *P*-values are generally valid for *P* < 0.001, especially for studies with 1000’s of foci.

To establish the validity of spatial homogeneity tests ($\mu_{j}^{X}=\mu_{0},\;\forall j=1,\ldots ,N$) for each of the 20 meta-analytic datasets, we compute *P*-values and create *P*–*P* plots. We compute 100 null realizations, each producing *N P*-values (one for each voxel), with the null expected $-{\;\text{log}\;}_{10}$  *P*-values ranging from $-{\;\text{log}\;}_{10}(N/(N+1))\approx 0$ to $-{\;\text{log}\;}_{10}(1/(N+1))=5.359$. To avoid the overplotting of 100 curves on the $-{\;\text{log}\;}_{10}$  *P*–*P* plots, for each ordered *P*-value index on the abscissa we compute the average and standard deviation (SD) of the 100 corresponding $-{\;\text{log}\;}_{10}$  *P*-values, plotting the mean and confidence bounds at ±1.96 SD. We rejected the null hypothesis of spatial homogeneity at a 5% significance level, and calculated the percentage of rejected voxels out of the 228, 483 voxels located within the brain. Since the *P*–*P* plots are very similar for each of the eight scenarios, we only display the results for the setting of CBMR with an NB model without study-level covariates, sampled with a model-based approach. Figure 3 shows the four representative $-{\;\text{log}\;}_{10}$  *P*–*P* plots (results for all 20 studies shown in Figure S11 in the Supplementary material), with identity (dashed diagonal line), 5% significance (dashed horizontal line) and the FDR 5% boundary (solid diagonal line); gray shaded areas plot the point-wise 95% prediction intervals. It shows that *P*-values $<0.05\approx 10^{-1.3}$ are valid, and extreme *P*-values can skew liberal; the worst affected cases are datasets with very few foci (e.g. analysis 14). In general, datasets with total foci counts less than 1000 show poor behavior.

Since multiple testing correction requires valid *P*-values far smaller than 0.05, we focus on controlling the FDR in these null simulations. None of the 20 datasets have valid FDR control (PP-plots or prediction intervals fall above the 5% Benjamini-Hochberg threshold). However, the PP plots generally show valid *P*-values $<10^{-3}$, and if we truncate *P*-values by replacing any *P*-value smaller than $10^{-3}$ with that value, we obtain valid (if conservative) FDR control (Table 2). This pragmatic approach could impact power, but empirical results (Section 4.2) suggest that the inferences based on truncated *P*-values remain sensitive.

**Table 2:** The percentage of invalid FDR control (before/after *P*-value truncated at $10^{-3}$) in 20 meta-analytic datasets over 100 realizations.

Dataset	Before	After	Dataset	Before	After
1. Social processing	44%	0%	2. PTSD	100%	0%
3. Substance use	26%	0%	4. Dementia	16%	0%
5. Cue reactivity	28%	0%	6. Emotion regulation	23%	0%
7. Decision making	18%	0%	8. Reward	43%	0%
9. Sleep deprivation	30%	0%	10. Naturalistic	22%	0%
11. Problem solving	26%	0%	12. Emotion	100%	0%
13. Cannabis use	63%	0%	14. Nicotine use	94%	0%
15. Frontal pole CBP	90%	0%	16. Face perception	19%	0%
17. Nicotine administration	54%	0%	18. Executive function	22%	0%
19. Finger tapping	22%	0%	20. n-Back	27%	0%

### 4.2 Results from 20 meta-analytic datasets

We first evaluate the goodness of fit among likelihood-based stochastic models (Poisson, NB and clustered NB model) via comparisons of maximized log-likelihood and AIC. As shown in Figures S12 and S13 in S4.3 of the Supplementary Material, CBMR with the NB model outperforms the other two likelihood-based stochastic models in every dataset. This is not surprising as the NB model is the only likelihood-based model that allows for the anticipated excess variance relative to Poisson at the voxel level; clustered NB is better than Poisson for the majority of these 20 meta-analytic datasets, but only by a small margin. It is conceivable that although a study-wise global dispersion parameter exists in the clustered NB model, CBMA data is just as well specified by a Poisson model at the voxel level. LRT comparison of nested models rejects the null Poisson model vs. NB for all datasets, with *P*-value less than $10^{-8}$; the Poisson null is rejected in favor of the clustered NB model for the majority of the 20 meta-analytic datasets (with *P*-value less than $10^{-8}$) (see Table S13 in Appendix S4.3 of the Supplementary Material).

For all methods we also use three metrics to assess model fit:

- **Relative Absolute Bias of Intensity Sum:** This metric compares the sum of CBMR estimated intensity over the space to the total number of observed foci counts within the dataset.
- **Relative Absolute Bias of Intensity Standard Deviation (SD) in the *x*, *y*, *z* directions:** This metric evaluates the SD of CBMR intensity estimation compared to the empirical distribution of foci counts in the dataset in all three directions.
- **Relative Absolute Bias of Variance at the voxel-wise level:** This metric measures the discrepancy between the asymptotic variance of the fitted CBMR model and empirical variance of foci counts in the dataset, calculated at each voxel and averaged over voxels with at least one foci.

These metrics were calculated for each of the 20 datasets and are presented using boxplots to illustrate the variability and distribution of the results.

**Fig. 4:**
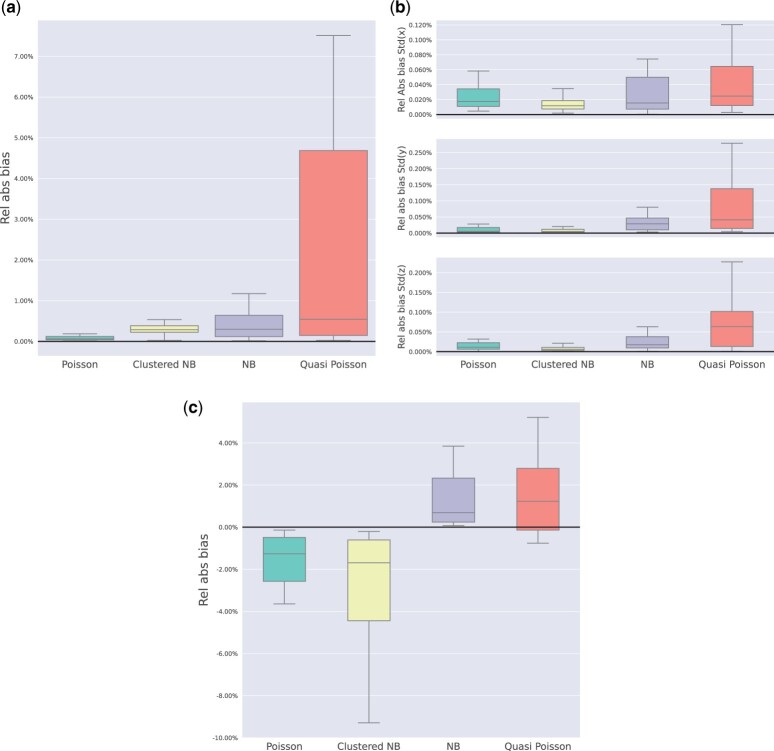
Results from bias-related model comparison criteria, fitted with four stochastic models on each of 20 meta-analytic datasets: (a) boxplot of relative absolute bias of estimated intensity sum (per study); (b) boxplot of relative absolute bias of estimated intensity SD in x,y,z directions (per study); (c) boxplot of relative absolute bias of voxelwise estimated intensity variance (per study).

Plots in Fig. 4a suggest that the four evaluated stochastic models (Poisson, NB, clustered NB and Quasi-Poisson model) produce consistently accurate estimates, with the median relative bias of estimated study-wise total foci count less than 1.0%, among which the Poisson model has the lowest median relative absolute bias (0.05%), across 20 meta-analytic datasets. However, all four stochastic models tend to slightly overestimate the study-wise total foci counts in these datasets. The Quasi-Poisson, in particular, shows a more variable relative absolute bias across the 20 datasets. The results in Fig. 4b suggest that the CBMR framework also provides an accurate estimation of standard variation (SD) of intensity across the *x*, *y*, *z* dimensions. The relative bias is controlled below 0.25% for all stochastic models in 20 meta-analytic datasets, and estimated intensity along the *x* axis are the most accurate (with the smallest SD bias). As shown in Fig. 4c, CBMR with the Poisson model and clustered NB model display a negative bias in variance which suggests that excess variance cannot be explained by the Poisson assumption. The study-specific over-dispersion modeled by the clustered NB is insufficient, as this model also has negative bias. Small relative bias is found in both NB and Quasi-Poisson model (with median 0.78% and 1.25%), with less variation in relative bias across multiple datasets with the NB model, which suggests both models are capable of dealing with excess variance in CBMA data.

Overall, we regard these evaluations as evidence that NB model is preferred. While it has slightly more bias in the study-wise total foci counts (Fig. 4a), its variance estimation is considerably more precise compared to the Poisson model (Fig. 4c).

### 4.3 Comparison with ALE

Activation likelihood estimation (ALE) is one of the widely used kernel-based CBMA methods. For each focus, ALE creates a map with a Gaussian kernel centred at the location, and then combines pairs of maps using the probability of a union of events rule (P(A∪B)=P(A)+P(B)−P(A∩B)) at each voxel (Turkeltaub et al. 2002). It appears to model the probability that one or more foci arise at a given voxel, conditional on the total number of foci over all studies.

**Fig. 5:**
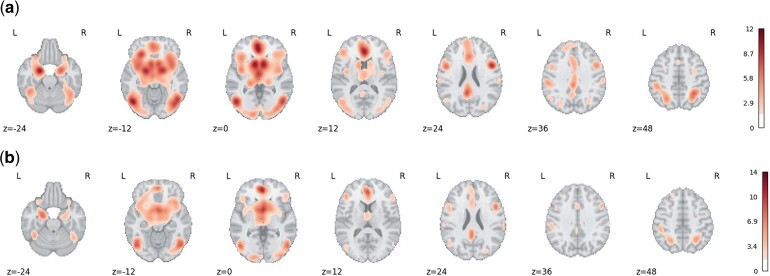
Activation maps (for significant uncorrected *P*-values, P≤5%, displayed as Z-scores) generated by ALE (with FWHM=14) and CBMR (with NB model) on the Cue Reactivity dataset with axial slices at z=−24,−12,0,12,24,36,48. Both methods identify similar regions for significant evidence against the null of spatial homogeneity. While ALE finds more significant voxels, it represents a fixed-effects type of analysis not directly comparable with CBMR inferences (see text). (a) Z-score map generated by ALE. (b) Z-score map generated by CBMR (with NB model).

We compared our CBMR results to ALE, conducting tests for spatial homogeneity across space with both approaches. For simplicity, we only demonstrate the comparison of detected activation regions on the Cue Reactivity dataset (total foci count of 6288) (Hill-Bowen et al. 2021), and only the z-value map generated by the CBMR with the NB model is presented here as a representative example. For comparison purposes, we show z-statistic values at all voxels significant at α=0.05 uncorrected in Fig. 5. Here, we choose FWHM=14 to obtain comparative spatial resolution between ALE and CBMR. Evidence for consistent activation is found in the left cerebral cortex, frontal orbital cortex, insular cortex, left and right accumbens, with exact activation regions differing slightly between ALE and CBMR, and ALE detecting more voxels.

Another criterion of consistency is the Dice Similarity Coefficient (DSC), the intersection of ALE and CMBR significant voxels divided by the average number of significant voxels. As shown in Table 3, ALE appears generally more sensitive than CBMR, regardless of foci counts in the datasets, though DSC varies from 71.89% to 80.33% on the datasets with more than 1200 foci counts, which demonstrates good similarity between the methods.

**Table 3:** Number of voxels in activation regions of ALE (FWHM=14) and CBMR (with the NB model), based on uncorrected *P*-values with 5% significance level, as well as Dice similarity coefficient in 20 meta-analytic datasets (Datasets are listed in an ascending order according to total number of foci).

Dataset	n_foci	$\left|AR_{\mathrm{CBMR}}\right|$	$\left|AR_{\mathrm{ALE}}\right|$	$\left|AR_{\mathrm{CBMR}}\cap AR_{\mathrm{ALE}}\right|$	DSC
14. Nicotine use	77	1312	12431	1154	17.79%
2. PTSD	154	6306	15866	5067	45.71%
13. Cannabis use	314	11841	18390	8235	54.48%
17. Nicotine administration	349	11546	18916	8028	52.71%
9. Sleep deprivation	454	10250	15461	5732	44.59%
20. n-Back	640	19404	31512	17627	69.24%
3. Substance use	657	19024	26477	13602	59.79%
19. Finger tapping	696	19067	33914	17939	67.72%
4. Dementia	1194	16244	30437	12464	53.41%
10. Naturalistic	1220	22328	29442	15344	59.28%
7. Decision making	1225	28284	36735	23372	71.89%
12. Emotion	2038	57698	67699	48847	77.91%
18. Executive function	2629	33848	46679	31698	78.73%
16. Face perception	2920	41682	53109	36710	77.45%
11. Problem solving	3043	38466	51315	34757	77.43%
5. Cue reactivity	3197	41242	52371	37301	79.69%
6. Emotion regulation	3543	36602	48157	31176	73.56%
1. Social processing	4934	48376	61136	40740	74.40%
15. Frontal pole CBP	9525	53165	65339	47595	80.33%
8. Reward	6791	43048	51721	37711	79.59%

ALE evaluates the experimental effect by testing probabilistic maps (generated by a Gaussian kernel) against the null hypothesis, CBMR estimates activation intensity and conducts hypothesis testing at the voxel level.

**Fig. 6:**
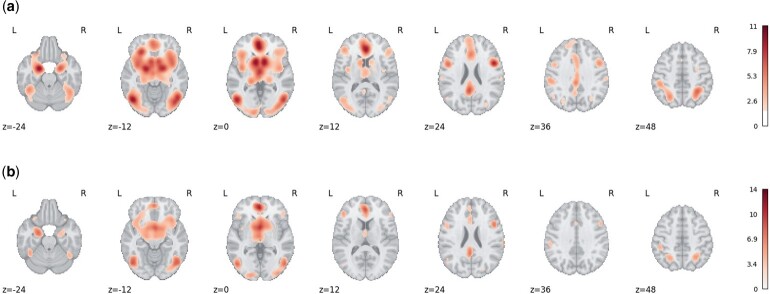
Activation maps (for significant FDR corrected *P*-values under 5% significance level, presented in Z-scores) generated by ALE and CBMR with FDR correction (by BH procedure) with truncated *P*-values of Cue Reactivity dataset. The figure is shown with axial slices at z=−24,−12,0,12,24,36,48. Under the null hypothesis of spatial homogeneity, activation regions with z-scores corresponding to corrected *P*-values below the significance level 0.05 are highlighted. (a) Z-score map generated by ALE. (b) Z-score generated by CBMR (with NB model).

Some researchers have proposed a stringent threshold (α=0.0001) on uncorrected *P*-values to reduce type I error (Turkeltaub et al. 2002), while a more principled approach is to control the false discovery rate (FDR) via Benjamini-Hochberg (BH) procedure. Figure 6 shows a comparison of results using a 5% FDR threshold, where CMBR (NB) *P*-values use a $10^{-3}$ truncation, and Table 4 shows a comparison of the number of detected voxels.

**Table 4:** Number of voxels in activation regions of ALE (FWHM=14) and CBMR (with the NB model), based on FDR corrected *P*-values (using BH procedure) with 5% significance level, as well as Dice similarity coefficient in 20 meta-analytic datasets (Datasets are listed in an ascending order according to total number of foci). Roughly, datasets with at least 1000 foci show reasonable similarity between ALE and CBMR.

Dataset	n_foci	$\left|AR_{\mathrm{CBMR}}\right|$	$\left|AR_{\mathrm{ALE}}\right|$	$\left|AR_{\mathrm{CBMR}}\cap AR_{\mathrm{ALE}}\right|$	DSC
14. Nicotine use	77	209	0	0	0.00%
2. PTSD	154	0	1201	0	0.00%
13. Cannabis use	314	313	152	17	7.31%
17. Nicotine administration	349	1338	943	522	45.77%
9. Sleep deprivation	454	176	0	0	0.00%
20. n-Back	640	11456	17725	10212	69.99%
3. Substance use	657	3145	2082	1225	46.87%
19. Finger tapping	696	12410	23837	11590	63.95%
4. Dementia	1194	5126	7931	3142	48.13%
10. Naturalistic	1220	4192	3241	1861	50.07%
7. Decision making	1225	15331	20468	12628	70.55%
18. Executive function	2629	26039	37797	24690	77.67%
16. Face perception	2920	28893	38193	25533	76.12%
11. Problem solving	3043	28221	39091	25675	76.29%
5. Cue reactivity	3197	30382	38847	27375	78.57%
6. Emotion regulation	3543	23388	31620	20056	72.92%
1. Social processing	4943	34317	45263	28555	71.76%
8. Reward	6791	33021	39743	28728	78.96%
15. Frontal pole CBP	9525	44030	55251	39594	79.76%
12. Emotion	22038	50480	57321	41918	77.77%

It is seen that CBMR generally detects fewer voxels than ALE (Table 4), however these two approaches are not directly comparable. In previous work (Samartsidis et al. 2017) it’s demonstrated that ALE behaves like a fixed-effect model, where significance can be driven by a tiny fraction of “real” studies mixed with purely noise studies. In contrast, with our model, heterogeneity can be captured by the NB excess variance term and make inferences more sceptical. As a result, direct comparisons of sensitivity seem akin to comparing the power of a fixed effects model (that neglects an important source of variation) to a mixed effects model, where the fixed effects model will always be more powerful by design. We also add that CBMR is grounded in a generative statistical model, accommodates study-level covariates and produces standard errors on interpretable parameters.

Instead of relative power, our goal here is to demonstrate that the detected activation regions produced by both methods are roughly consistent. For this purpose, we found that the DSC varies between 70.55% and 79.76% for datasets with more than 1225 foci counts, indicating consistency of activation regions between ALE and CBMR approach after FDR correction.

### 4.4 Effect of study-level covariates

Here we demonstrate how CBMR, unlike ALE, can estimate the effect of study-level covariates. We integrate two study-level covariates, study-wise (square root) sample size and year of publication (after centring and standardisation) into the CBMR framework on each of the 20 meta-analytic datasets. We find, for example, on Cue Reactivity dataset, the year of publication is not significant (Z=−0.6880,p=0.4915), while sample size is significant ($Z=6.1454,p<10^{-8}$); interpreting the *γ* parameter for sample size finds that a doubling of sample size results in an expected 26.15% increase in the study-wise spatial intensity (see Table S14 for *P*-values and *Z*-scores of study-level covariates on each of the 20 meta-analytic datasets).

## 5 Discussion

In this work we have presented a meta-regression framework with a spatial model as a general approach for CBMA data, where we have considered multiple stochastic models and allowed for study-level covariates (e.g. sample size and year of publication). Our approach uses spline parameterization to model the smooth spatial distribution of activation foci, and fits a generalized linear model with different variants of voxelwise (Poisson model, NB model and Quasi-Poisson model) or study-wise (Clustered NB model) statistical distributions. Our approach is a computationally efficient alternative to previous Bayesian spatial regression models, providing the flexibility and interpretability of a regression model while jointly modeling all of space. For comparison, using the Cue Reactivity dataset as an example, the implementation of Bayesian log-Gaussian Cox process regression required approximately 30 hours on an NVIDIA Tesla K20c GPU card Samartsidis et al. (2019), in contrast to approximately 537.52 seconds (about 9 minutes) required for CBMR with the NB model on an Intel Xeon Gold 6340R CPU. Furthermore, grounded in a generalized linear model, we believe that our meta-regression framework is more comprehensible to practitioners, relative to inference on the spatial posterior intensity function.

Through simulations on synthetic data (with simulated foci counts analogous to those in each of 20 meta-analytic datasets), we demonstrated valid FDR control for the spatial homogeneity null hypothesis after a truncation of *P*-values below $10^{-3}$. According to 20 meta-analytic datasets, we found that the NB model is the most accurate stochastic model in model comparisons via LRT and AIC, as well as having the smallest relative bias in both mean and variance of intensity estimation (per study), while the Poisson and clustered NB model cannot explain the over-dispersion observed in foci counts. Meanwhile, we also compared the findings of activation regions from both the ALE and CBMR approach, and justified the validity and robustness of CBMR, especially on the datasets with relatively high foci counts, e.g. datasets with at least 1000 total foci.

There are a few limitations in our work. Here we have only considered a single group of studies. In future work, we plan to extend our method to estimate the spatial intensity function of multiple groups (e.g. multiple types of stimuli within a cognitive task), so that we can investigate the consistency and difference in activation regions through group comparison. Additionally, our current analysis is limited to the global effects of study-level covariates, a pragmatic decision given common application with 10’s-100’s of studies. We recognize, however, that this approach might not be appropriate in cases where there are significant spatial variations in covariate effects. Ideally we would add a basis function to express each covariate effect, though this would likely be infeasible without many 1000’s of studies. Alternatively we could use a coarse parcellation (e.g. 3-6 regions) and allow parcel-specific regression coefficients for each region.

We are currently not using regularization term on spatial regression coefficients of CBMR. Initially we considered a Firth-type penalty which indeed guarantees convergent estimates (especially in brain regions without any foci) and removes the first-order asymptotic bias term of maximum likelihood estimates, but we found it also causes significant overestimation of intensity at the edge of brain mask. The edge effect induced by Firth-type penalty relates to the structure of the Jeffreys prior and higher variance associated with edge and corner basis elements. However, it’s plausible to consider regularizing likelihood functions with alternative penalty terms (e.g. *L*_1_ or *L*_2_ norm) in the future, though requiring hyper-parameter tuning. We estimate the variance of voxel-wise spatial intensity using the covariance of spatial regression coefficients found by inverting the Fisher Information matrix. This can be numerically unstable because the dimension of Fisher Information matrix is large (there are hundreds or even thousands elements in spline bases), and it might even be numerically singular for datasets with low foci counts since most voxels have near-zero intensity estimation. We have tried many approaches to improve numerical stability, including adding an extremely small epsilon ($10^{-6}$) or 1% of the largest diagonal element on the diagonal of the Fisher Information matrix, or computing the Fisher Information assuming the null hypothesis of homogeneity is true. However, all of these efforts produced underestimation of the variance of voxel-wise spatial intensity and led to invalid *P*-values. In future work, we might consider non-parametric methods to estimate the covariance of spatial regression coefficient instead of the inverse of Fisher Information, or add a regularization term on B-spline roughness to avoid very negative spatial regression coefficients.

Another important direction is a combined IBMA-CBMA analysis, were we extend our model to include continuous effect size maps. One possible approach is to combine separate coordinate and intensity models using Markov melding in a fully Bayesian framework for joining probabilistic sub-models. In this approach, evidence from each different source is specified in each sub-model, and the sub-models are joined while preserving all information and uncertainty (Goudie et al. 2019). Such an approach might enrich the inference obtained from CBMR by integrating the magnitude of CBMA activation or even image-based meta-analysis data.

Another direction of interest is investigating the variability caused by different meta-analysis pipelines. This consideration is important, as we have observed significant variation in activation regions due to the different sensitivity in analysis pipelines in each study. In fact, the CBMR’s study-level covariates already allow it to accommodate variations in analysis pipelines by including the specific pipeline used as a study-level covariates and understanding its impact at a global level.

Finally, another direction to consider is a zero-inflated stochastic model (e.g. Poisson or NB model) as the current datasets only consist of studies with at least one focus, there might be inflated zero foci count than observed. Excess zeros are separated and modeled independently in zero-inflated models, which might provide a more accurate approximation for low-rate Binomial data, as was found useful when modeling image-wise total foci counts (Samartsidis et al. 2020).

## Supplementary Material

kxae024_Supplementary_Data

## Data Availability

Implementation in the form of Python and Pytorch code can be found in Github repository. CBMR framework has also been implemented and integrated into NiMARE python package
