# Supplementary material for: Neuroimaging meta regression for coordinate based meta analysis data with a spatial model
Source: Biostatistics. 2024 Jul 13;25(4):1210–32. doi: 10.1093/biostatistics/kxae024 (PMC11471956; doi:10.1093/biostatistics/kxae024)
Supplement: kxae024_Supplementary_Data [file kxae024_supplementary_data.pdf]

Biostatistics (2024), **0**, 0, *pp.* 1–**??**
doi:10.1093/biostatistics/output

Neuroimaging Meta Regression for Coordinate Based Meta Analysis Data with a Spatial Model: Supplementary materials

Yifan Yu[1], Rosario Pintos Lobo[2], Michael Cody Riedel[3], Katherine Bottenhorn[4], Angela R. Laird[5], Thomas E. Nichols[1,6*]

[1] *Oxford Big Data Institute, University of Oxford, Oxford, UK*

[2] *Department of Psychology, Florida International University, Miami, FL, USA*

[3] *Department of Physics, Florida International University, Miami, FL, USA*

[4] *Department of Population and Public Health Sciences, University of Southern California, Los Angeles, CA, USA*

[5] *Center for Imaging Science, Florida International University, Miami, FL, USA*

[6] *Wellcome Centre for Integrative Neuroimaging, FMRIB, Nuffield Department of Clinical Neurosciences, Oxford, UK*

thomas.nichols@bdi.ox.ac.uk

*To whom correspondence should be addressed.

© The Author 2024. Published by Oxford University Press. All rights reserved. For permissions, please e-mail: journals.permissions@oup.com

APPENDIX

S1. Detailed derivation of stochastic models

S1.1 *Poisson model*

We assert that the sum of two independent Poisson random variables is also Poisson. Let $X \sim \text{Poi}(\lambda_1)$ and $Y \sim \text{Poi}(\lambda_2)$ be two independent random variables, and $Z = X + Y$, then,

$$\begin{aligned} P(Z=n) = P(X+Y=N) &= \sum_{k=-\infty}^{\infty} P(X=k)P(Y=n-K) \\ &= \sum_{k=0}^{n} P(X=k)P(Y=n-k) \\ &= \sum_{k=0}^{n} e^{-\lambda_1}\frac{\lambda_1^k}{k!}e^{-\lambda_2}\frac{\lambda_2^{n-k}}{(n-k)!} \\ &= e^{-(\lambda_1+\lambda_2)}\sum_{k=0}^{n}\frac{\lambda_1^k\lambda_2^{n-k}}{k!(n-k)!} \\ &= \frac{e^{-(\lambda_1+\lambda_2)}}{n!}\sum_{k=0}^{n}\frac{n!}{k!(n-k)!}\lambda_1^k\lambda_2^{n-k} \\ &= \frac{e^{-(\lambda_1+\lambda_2)}}{n!}(\lambda_1+\lambda_2)^n \end{aligned} \tag{A.1}$$

Therefore, $Z = X + Y \sim \text{Poi}(\lambda_1 + \lambda_2)$ is also a Poisson variable. The conclusion can be extended further: the sum of multiple Poisson random variables ($\text{Poi}(\lambda_i), i = 1, \cdots, n$) also follows a Poisson distribution, with the parameter $\lambda = \sum_{i=1}^{n} \lambda_i$.

Hence, under the assumption of independence of counts across studies, we believe that the likelihood function is exactly the same if we model the voxelwise total foci count over studies (with length-N) instead of voxelwise foci count for each study (with expanded length-(NM)). This reformulation can simplify the computation of the log-likelihood function and reduce the dimensionality of statistics (never larger than M or N in dimension).

S1.2 *Negative Binomial (Poisson-Gamma) Model*

In this section, we describe the formulation of the NB distribution in more detail. Based on the assumption of NB (Poisson-Gamma) model, there is a single parameter α, which indicates variance in excess of the Poisson model. For voxel j in study i, the voxelwise mean of intensity λ_{ij} follows a Gamma distribution with mean $\mathbb{E}(\lambda_{ij}) = \mu_{ij}$ and variance $\text{Var}(\lambda_{ij}) = \alpha\mu_{ij}^2$

$$\lambda_{ij} \sim \text{Gamma}(\alpha^{-1}, \frac{\alpha^{-1}}{\mu_{ij}}) \Rightarrow \mathbb{E}(\lambda_{ij}) = \mu_{ij}, \text{Var}(\lambda_{ij}) = \alpha\mu_{ij}^2$$

And $Y_{ij}|\lambda_{ij}$ follows a Poisson distribution with conditional mean $\mathbb{E}(Y_{ij}|\lambda_{ij}) = \lambda_{ij}$

$$Y_{ij}|\lambda_{ij} = \text{Poisson}(\lambda_{ij}) \Rightarrow P(Y_{ij}|\lambda_{ij} = k) = \frac{\lambda_{ij}^k e^{-\lambda_{ij}}}{k!}$$

which gives rise to marginal probability of Y_{ij}

$$\begin{aligned}
P(Y_{ij} = y_{ij}) &= \int_{\lambda_{ij}} P(Y_{ij}|\lambda_{ij})P(\lambda_{ij})d\lambda_{ij} = \int_{\lambda_{ij}=0}^{\infty} \frac{\lambda_{ij}^{y_{ij}} e^{-\lambda_{ij}}}{y_{ij}!} \frac{(\frac{1}{\alpha\mu_{ij}})^{\frac{1}{\alpha}}}{\Gamma(\frac{1}{\alpha})} \lambda_{ij}^{\frac{1}{\alpha}-1} e^{-\frac{\lambda_{ij}}{\alpha\mu_{ij}}} d\lambda_{ij} \\
&= \frac{\frac{1}{\alpha\mu_{ij}}^{\frac{1}{\alpha}}}{y_{ij}!\Gamma(\frac{1}{\alpha})} \int_{\lambda_{ij}=0}^{\infty} \lambda_{ij}^{y_{ij}} e^{-\lambda_{ij}} \lambda_{ij}^{\frac{1}{\alpha}-1} e^{-\frac{\lambda_{ij}}{\alpha\mu_{ij}}} d\lambda_{ij} = \frac{\frac{1}{\alpha\mu_{ij}}^{\frac{1}{\alpha}}}{y_{ij}!\Gamma(\frac{1}{\alpha})} \frac{\Gamma(y_{ij} + \frac{1}{\alpha})}{(\frac{1}{\alpha\mu_{ij}} + 1)^{y_{ij}+\frac{1}{\alpha}}} \\
&= \frac{\Gamma(y_{ij} + \frac{1}{\alpha})}{\Gamma(y_{ij} + 1)\Gamma(\frac{1}{\alpha})} \left(\frac{\frac{1}{\alpha\mu_{ij}}}{\frac{1}{\alpha\mu_{ij}} + 1}\right)^{\frac{1}{\alpha}} \left(\frac{1}{\frac{1}{\alpha\mu_{ij}} + 1}\right)^{y_{ij}} = \frac{\Gamma(y_{ij} + \alpha^{-1})}{\Gamma(y_{ij} + 1)\Gamma(\alpha^{-1})} \left(\frac{1}{1 + \alpha\mu_{ij}}\right)^{\alpha^{-1}} \left(\frac{\alpha\mu_{ij}}{1 + \alpha\mu_{ij}}\right)^{y_{ij}}
\end{aligned}$$

which satisfies the mathematical form of probability density function of the NB model, $Y_{ij} \sim NB(\alpha^{-1}, \frac{\mu_{ij}}{\alpha^{-1}+\mu_{ij}})$, with mean $\mathbb{E}[Y_{ij}] = \mu_{ij}$ and variance $\mathbb{V}(Y_{ij}) = \mu_{ij} + \alpha\mu_{ij}^2$.

S1.3 *Moment Matching Approach*

For the purpose of approximating the sum of multiple independent NB random variables, we approximate a sum of NB variates with a NB distribution by moment matching (mean and variance). Suppose the voxelwise count in each individual study is $Y_{ij} \sim \text{NB}(\alpha^{-1}, \frac{\mu_{ij}}{\mu_{ij}+\alpha^{-1}})$, and

α is a global dispersion parameter. Using the independence of studies at voxel j,

$$\begin{cases} \mathbb{E}(Y_{\bullet,j}) = \sum_{i=1}^{M} \mathbb{E}(Y_{ij}) = \sum_{i=1}^{M} \mu_{ij} \\ \mathbb{V}(\mathbb{E}(Y_{\bullet,j})) = \sum_{i=1}^{M} \mathrm{Var}(Y_{ij}) = \sum_{i=1}^{M} \mu_{ij} + \sum_{i=1}^{M} \alpha\mu_{ij}^2 \end{cases}$$

To ensure that the proposed NB distribution ($Y_{\bullet,j} \sim NB(r', p')$) matches the mixture of NB distributions, with regard to both mean and variance, we need

$$\begin{cases} \mathbb{E}(Y_{\bullet,j}) = \sum_{i=1}^{M} \mu_{ij} \\ \mathrm{Var}(Y_{\bullet,j}) = \sum_{i=1}^{M} \mu_{ij} + \sum_{i=1}^{M} \alpha\mu_{ij}^2 \end{cases} \Rightarrow \begin{cases} p' = \frac{\sum_{i=1}^{M} \mu_{ij}^2}{\alpha^{-1}\sum_{i=1}^{M} \mu_{ij} + \sum_{i=1}^{M} \mu_{ij}^2} \\ r' = \frac{(\sum_{i=1}^{M} \mu_{ij})^2}{\alpha \sum_{i=1}^{M} \mu_{ij}^2} \end{cases}$$

Therefore, the approximated NB distribution of the sum of foci count at voxel j is, $Y_{\bullet,j} \sim \mathrm{NB}\left(\frac{(\sum_{i=1}^{M} \mu_{ij})^2}{\alpha \sum_{i=1}^{M} \mu_{ij}^2}, \frac{\sum_{i=1}^{M} \mu_{ij}^2}{v \sum_{i=1}^{M} \mu_{ij} + \sum_{i=1}^{M} \mu_{ij}^2}\right)$, with excess variance in the NB approximation α',

$$\frac{1}{\alpha'} = \frac{(\sum_{i=1}^{M} \mu_{ij})^2}{\alpha \sum_{i=1}^{M} \mu_{ij}^2} \Rightarrow \alpha' = \frac{\sum_{i=1}^{M} \mu_{ij}^2}{(\sum_{i=1}^{M} \mu_{ij})^2} \alpha$$

.

S1.4 *Evaluating the effectiveness of moment matching approach*

To evaluate the effectiveness of the moment matching approach and to substantiate its application to CBMR with the NB model, we now incorporate simulation experiments to provide empirical evidence supporting this application.

We conducted a univariate simulation with $N = 10000$ studies, where the true rate is homogeneous but the dispersion parameter is shared. Specifically, each study i has mean $\mu_i = \mu_0 \cdot \frac{10i}{N}$, $\mu_0 = 10^{-3}$, and variance $\mu_i + \alpha\mu_i^2$, $\alpha = 0.5$, generating Y_i, $i = 1, \cdots, N$,

$$Y_i \sim NB(\mu_i, \alpha), \quad \mu_i = \mu_0 \cdot \frac{10i}{N} \tag{A.2}$$

Our approximate approach for modelling this data is to assume

$$\sum_i Y_i \sim NB(\mu, \alpha') \tag{A.3}$$

where $\mu = \sum\limits_i \mu_i = \mu_0 \cdot \frac{10(N+1)}{2}$, and α' is given by $(\sum\limits_i \mu_i^2)/(\sum\limits_i \mu_i)^2\alpha$. Like in our full CBMR model, here we use Maximum Likelihood (ML) to estimate μ and α, and we construct large-sample standard errors for μ using Fisher's information. Using 1000 Monte Carlo (MC) realisations, we compute the MC standard deviation of $\hat{mu}$ and compare it to the large sample standard errors (inverse Fisher's information). Our analysis revealed that, with 1000 Monte Carlo realisations, the MC standard deviation of $\hat{\mu}$ is 7.1000. In comparison, the standard error, estimated using the Fisher information from the log-likelihood function, is 7.0780. The relative bias is 0.3098%, which strongly supports the accuracy of the standard error estimates for $\hat{\mu}$ in the moment matching approach.

The figure S7 presented below further justifies the accuracy of the moment matching approach. As demonstrated, the estimate of the mean sum across all studies ($\mu = \sum_i \mu_i$) can be effectively parameterised using μ_0 alone. It is evident that the log-likelihood functions of the moment matching approach and the exact log-likelihood have consistent shapes, although they are on different value scales. The Maximum likelihood estimates (MLEs) for both methods are remarkably close to their true value of 10^{-3}. This indicates that the MLE for the mean sum of the moment matched Negative Binomial (NB) distribution is precise.

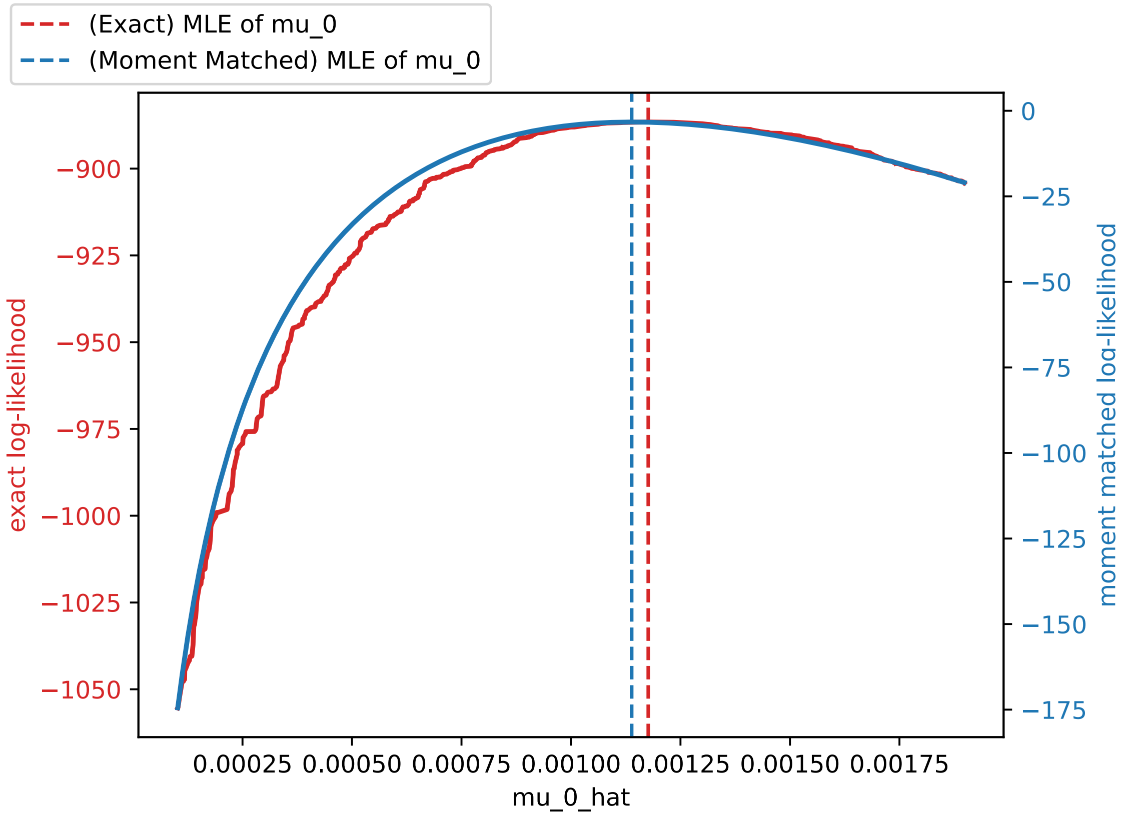

Figure S7: Exact log-likelihood functions of NB distribution and its moment matching approximation

S1.5 *Two-stage hierarchy Poisson-Gamma model*

In this section, we propose a two-stage hierarchy Poisson-Gamma model, which regards the random (Gamma) effect as a latent characteristic of each study, instead of independent voxelwise effects. The name “two-stage hierarchy Poisson-Gamma model” comes from the modelling procedure: consider the clustered count data Y_{ij}, $i = 1, \cdots, M$ (number of studies), $j = 1, \cdots, N$ (number of voxels). Draw λ_i from a Gamma distribution with mean 1 and variance α.

$$\lambda_i \sim \text{Gamma}(\alpha^{-1}, \alpha^{-1}) \Rightarrow \mathbb{E}(\lambda_i) = 1, \mathbb{V}(\lambda_i) = \alpha,$$

$$f(\lambda_i) = \frac{\frac{1}{\alpha}^{\alpha^{-1}}}{\Gamma(\alpha^{-1})} \lambda_i^{\alpha^{-1}-1} e^{-\alpha^{-1}\lambda_i}$$

For each study i, for each voxel j, $Y_{ij}|\lambda_i$ are drawn from a Poisson distribution with mean $\lambda_i\mu_{ij}$, where μ_{ij} is the spatial mean parameterized by some β (B-spline basis coefficients).

$$Y_{ij}|\lambda_i \sim \text{Poisson}(\lambda_i\mu_{ij}) \Rightarrow P(Y_{ij}|\lambda_i = k) = \frac{(\lambda_i\mu_{ij})^k e^{-\lambda_i\mu_{ij}}}{k!}$$

Therefore, the marginal probability of the foci count Y_{ij} is,

$$\begin{aligned}
P(Y_{ij} = y_{ij}) &= \int\limits_{\lambda_i} P(Y_{ij}|\lambda_i)P(\lambda_i)d\lambda_i = \int\limits_{\lambda_i=0}^{\infty} \frac{(\mu_{ij}\lambda_i)^{y_{ij}} e^{-\mu_{ij}\lambda_i}}{y_{ij}!} \frac{\frac{1}{\alpha}^{\alpha^{-1}}}{\Gamma(\alpha^{-1})} \lambda_i^{\alpha^{-1}-1} e^{-\alpha^{-1}\lambda_i} d\lambda_i \\
&= \frac{\mu_{ij}^{y_{ij}} \frac{1}{\alpha}^{\alpha^{-1}}}{\Gamma(\alpha^{-1}) y_{ij}!} \int\limits_{\lambda_i=0}^{\infty} \lambda_i^{y_{ij}+\alpha^{-1}-1} e^{-\lambda_i(\mu_{ij}+\alpha^{-1})} d\lambda_i = \frac{\mu_{ij}^{y_{ij}} \frac{1}{\alpha}^{\alpha^{-1}}}{\Gamma(\alpha^{-1}) y_{ij}!} \frac{\Gamma(y_{ij}+\alpha^{-1})}{(\mu_{ij}+\alpha^{-1})^{y_{ij}+\alpha^{-1}}} \\
&= \frac{\Gamma(y_{ij}+\alpha^{-1})}{\Gamma(y_{ij}+1)\Gamma(\alpha^{-1})} \left(\frac{\mu_{ij}}{\mu_{ij}+\alpha^{-1}}\right)^{y_{ij}} \left(\frac{\alpha^{-1}}{\mu_{ij}+\alpha^{-1}}\right)^{\alpha^{-1}}
\end{aligned}$$

Therefore, the marginal distribution of foci count follows a NB distribution, $Y_{ij} \sim \text{NB}(\alpha^{-1}, \frac{\mu_{ij}}{\mu_{ij}+\alpha^{-1}})$, with mean $\mathbb{E}(Y_{ij}) = \mu_{ij}$ and variance $\text{Var}(Y_{ij}) = \mu_{ij} + \alpha\mu_{ij}^2$.

S1.6 *Covariance structure in Clustered NB model*

The two-stage hierarchical clustered NB model also introduces a covariance structure between foci within a study. Specifically, the covariance of the number of count in voxel j and voxel j' (Y_{ij} and $Y_{ij'}$) in study i, modelled by the clustered NB model is,

$$\begin{aligned}
\mathbb{E}[Y_{ij}Y_{ij'}] &= \mathbb{E}_{\lambda_i}\left[\mathbb{E}[Y_{ij}Y_{ij'}|\lambda_i]\right] = \mathbb{E}_{\lambda_i}\left[\mathbb{E}[Y_{ij}|\lambda_i]\mathbb{E}[Y_{ij'}|\lambda_i]\right] = \mathbb{E}_{\lambda_i}\left[(\lambda_i\mu_{ij})(\lambda_i\mu_{ij'})\right] \\
&= \mu_{ij}\mu_{ij'} \int_{\lambda_i} \lambda_i^2 f(\lambda_i) d\lambda_i = \mu_{ij}\mu_{ij'} \int_{\lambda_i} \frac{\frac{1}{\alpha}^{\alpha^{-1}}}{\Gamma(\alpha^{-1})} \lambda_i^{\alpha^{-1}+1} e^{-\alpha^{-1}\lambda_i} d\lambda_i \\
&= \mu_{ij}\mu_{ij'} \frac{\frac{1}{\alpha}^{\alpha^{-1}}}{\Gamma(\alpha^{-1})} \alpha^{\alpha^{-1}+1} \alpha \int_{\lambda_i} (\frac{1}{\alpha}\lambda_i)^{(\alpha^{-1}+1)} e^{\alpha^{-1}\lambda_i} d(\alpha^{-1}\lambda_i) \\
&= \mu_{ij}\mu_{ij'} \alpha^2 \frac{1}{\Gamma(\alpha^{-1})} \Gamma(\alpha^{-1}+2) = \mu_{ij}\mu_{ij'} \alpha^2 \alpha^{-1}(\alpha^{-1}+1) \\
&= (1+\alpha)\mu_{ij}\mu_{ij'}
\end{aligned}$$

For different voxels j and j' within a same study i,

$$\mathrm{Cov}(Y_{ij}Y_{ij'}) = \mathbb{E}[Y_{ij}Y_{ij'}] - \mathbb{E}[Y_{ij}]\mathbb{E}[Y_{ij'}] = (1+\alpha)\mu_{ij}\mu_{ij'} - \mu_{ij}\mu_{ij'} = \alpha\mu_{ij}\mu_{ij'}$$

While for different studies i and i',

$$\mathrm{Cov}(Y_{ij}Y_{i'j'}) = 0$$

Now, we will look at the total log-likelihood function of the clustered NB model, using dependence between studies. Let $Y_{i,\cdot} = \sum_{j=1}^{N} Y_{ij}$ be the sum of foci within study i regardless of location. The joint probability for the number of counts Y_{ij} ($\forall j = 1, \cdots, N$) from the ith study is,

$$f(Y_{i1}, Y_{i2}, \cdots, Y_{iN}) = \int_{\lambda_i} f(Y_{i1}, Y_{i2}, \cdots, Y_{iN}|\lambda_i) f(\lambda_i) d\lambda_i = \int_{\lambda_i} \prod_{j=1}^{N} f(Y_{ij}|\lambda_i) f(\lambda_i) d\lambda_i$$

$$= \int_{\lambda_i} \prod_{j=1}^{N} \frac{(\mu_{ij}\lambda_i)^{Y_{ij}} e^{-\mu_{ij}\lambda_i}}{Y_{ij}!} \frac{\frac{1}{\alpha}^{\alpha^{-1}}}{\Gamma(\alpha^{-1})} \lambda_i^{\alpha^{-1}-1} e^{-\alpha^{-1}\lambda_i} d\lambda_i$$

$$= \frac{\frac{1}{\alpha}^{\alpha^{-1}} \prod_{j=1}^{N} \mu_{ij}^{Y_{ij}}}{\Gamma(\alpha^{-1}) \prod_{j=1}^{N} Y_{ij}!} \int_{\lambda_i} \exp\{-\lambda_i(\alpha^{-1} + \sum_{j=1}^{N} \mu_{ij})\} \lambda_i^{\sum_{j=1}^{N} Y_{ij} + \alpha^{-1} - 1} d\lambda_i$$

$$= \frac{\frac{1}{\alpha}^{\alpha^{-1}} \prod_{j=1}^{N} \mu_{ij}^{Y_{ij}}}{\Gamma(\alpha^{-1}) \prod_{j=1}^{N} Y_{ij}!} \int_{\lambda_i} \exp\{-\lambda_i(\alpha^{-1} + \mu_{i,\cdot})\} \lambda_i^{Y_{i,\cdot} + \alpha^{-1} - 1} d\lambda_i$$

$$= \frac{\frac{1}{\alpha}^{\alpha^{-1}} \prod_{j=1}^{N} \mu_{ij}^{Y_{ij}}}{\Gamma(\alpha^{-1}) \prod_{j=1}^{N} Y_{ij}!} \frac{1}{(\alpha^{-1} + \mu_{i,\cdot})^{Y_{i,\cdot} + \alpha^{-1} - 1}} \frac{1}{\alpha^{-1} + \mu_{i,\cdot}} \cdot$$

$$\int_{\lambda_i} \exp\{-\lambda_i(\alpha^{-1} + \mu_{i,\cdot})\} [\lambda_i(\alpha^{-1} + \mu_{i,\cdot})]^{Y_{i,\cdot} + \alpha^{-1} - 1} d[\lambda_i(\alpha^{-1} + \mu_{i,\cdot})]$$

$$= \frac{\prod_{j=1}^{N} \mu_{ij}^{Y_{ij}}}{\Gamma(\alpha^{-1}) \prod_{j=1}^{N} Y_{ij}!} \frac{\frac{1}{\alpha}^{\alpha^{-1}}}{(\alpha^{-1} + \mu_{i,\cdot})^{Y_{i,\cdot} + \alpha^{-1}}} \Gamma(Y_{i,\cdot} + \alpha^{-1})$$

$$= \frac{\Gamma(Y_{i,\cdot} + \alpha^{-1}) \frac{1}{\alpha}^{\alpha^{-1}}}{\Gamma(\alpha^{-1}) \prod_{j=1}^{N} Y_{ij}!} (\alpha^{-1} + \mu_{i,\cdot})^{-(Y_{i,\cdot} + \alpha^{-1})} \exp\left[\sum_{j=1}^{N} Y_{ij} \log(\mu_{ij})\right]$$

It gives rise to the log-likelihood function for study i,

$$\log f(Y_{i1},\cdots,Y_{iN}) = \alpha^{-1}\log(\alpha^{-1}) + \log\Gamma(Y_{i,\bullet}+\alpha^{-1}) - \log\Gamma(\alpha^{-1}) - \sum_{j=1}^{N}\log Y_{ij}! \\ -(Y_{i,\bullet}+\alpha^{-1})\log(\alpha^{-1}+\mu_{i,\bullet}) + \sum_{j=1}^{N} Y_{ij}\log(\mu_{ij})$$

Therefore, using the independence between study i and i' $(i \neq i')$, the log-likelihood of $Y_{ij}(\forall i = 1,\cdots,M, j=1,\cdots,N)$ across all studies and voxels is,

$$\begin{aligned} l(\beta,\alpha) &= \sum_{i=1}^{M}\log[f(Y_{i1},Y_{i2},\cdots,Y_{iN})] \\ &= M\alpha^{-1}\log(\alpha^{-1}) - M\log\Gamma(\alpha^{-1}) + \sum_{i=1}^{M}\log\Gamma(Y_{i,\bullet}+\alpha^{-1}) - \sum_{i=1}^{M}\sum_{j=1}^{N}\log Y_{ij}! \\ &- \sum_{i=1}^{M}(Y_{i,\bullet}+\alpha^{-1})\log(\alpha^{-1}+\mu_{i,\bullet}) + \sum_{i=1}^{M}\sum_{j=1}^{N} Y_{ij}\log(\mu_{ij}) \end{aligned}$$

Using the assumption that $Y_{ij}=0$ or 1, so that $\log(Y_{ij}!)=0$ and $\mu_{ij}=\mu_j^X\mu_i^Z$,

$$\begin{aligned} l(\beta,\alpha) &= M\alpha^{-1}\log(\alpha^{-1}) - M\log\Gamma(\alpha^{-1}) + \sum_{i=1}^{M}\log\Gamma(Y_{i,\bullet}+\alpha^{-1}) - \sum_{i=1}^{M}(Y_{i,\bullet}+\alpha^{-1})\log(\alpha^{-1}+\mu_{i,\bullet}) + \sum_{i=1}^{M}\sum_{j=1}^{N} Y_{ij}\log(\mu_{ij}) \\ &= M\alpha^{-1}\log(\alpha^{-1}) - M\log\Gamma(\alpha^{-1}) + \sum_{i=1}^{M}\log\Gamma(Y_{i,\bullet}+\alpha^{-1}) - \sum_{i=1}^{M}(Y_{i,\bullet}+\alpha^{-1})\log(\alpha^{-1}+\mu_{i,\bullet}) \\ &+ \left(\sum_{j=1}^{N}\sum_{i=1}^{M} Y_{ij}\right)\left(\sum_{k=1}^{P} X_{jk}\beta_k\right) \\ &= M\alpha^{-1}\log(\alpha^{-1}) - M\log\Gamma(\alpha^{-1}) + \sum_{i=1}^{M}\log\Gamma(Y_{i,\bullet}+\alpha^{-1}) - \sum_{i=1}^{M}(Y_{i,\bullet}+\alpha^{-1})\log(\alpha^{-1}+\mu_{i,\bullet}) \\ &+ \left[\sum_{j=1}^{N} Y_{\bullet,j}\sum_{k=1}^{P} X_{jk}\beta_k\right] \\ &= M\alpha^{-1}\log(\alpha^{-1}) - M\log\Gamma(\alpha^{-1}) + \sum_{i=1}^{M}\log\Gamma(Y_{i,\bullet}+\alpha^{-1}) - \sum_{i=1}^{M}(Y_{i,\bullet}+\alpha^{-1})\log(\alpha^{-1}+\mu_{i,\bullet}) + Y_{\bullet,}^{\top}\log(\mu^X) \end{aligned}$$

S2. Deterministic model

S2.1 Model factorisation: Poisson model

We consider model factorisation to replace the full $(MN) - vector$ of foci counts with sufficient statistics (the dimension of which is not larger than M or N). Following the total log-likelihood function in Equation (2.5),

$$
\begin{aligned}
l &= \sum_{i=1}^{M}\sum_{j=1}^{N}[Y_{ij}\log(\mu_{ij}) - \mu_{ij} - \log(y_{ij}!)] = \sum_{i=1}^{M}\sum_{j=1}^{N} Y_{ij}\log\mu_{ij} - \sum_{i=1}^{M}\sum_{j=1}^{N}\mu_{ij} - 0 \\
&= \left(\sum_{i=1}^{M}\sum_{j=1}^{N} Y_{ij}\right)\left(\sum_{k=1}^{P} X_{jk}\beta_k + \sum_{s=1}^{R} Z_{is}\gamma_s\right) - \sum_{i=1}^{M}\sum_{j=1}^{N}\mu_j^X\mu_i^Z \\
&= \left[\sum_{j=1}^{N} Y_{\bullet,j}\sum_{k=1}^{P} X_{jk}\beta_{gk}\right] + \sum_{i=1}^{M} Y_{i,\bullet}\sum_{s=1}^{R} Z_{is}\gamma_s - \left[\sum_{j=1}^{N}\mu_j^X\right]\left[\sum_{i=1}^{M}\mu_i^Z\right] \\
&= \left[\sum_{j=1}^{N} Y_{\bullet,j}\log\mu_j^X\right] + \sum_{i=1}^{M} Y_{i,\bullet}\log\mu_i^Z - \left[\mathbf{1}^\top\mu^X\right]\left[\mathbf{1}^\top\mu^Z\right] \\
&= Y_{\bullet,}^\top\log(\mu^X) + Y_{,\bullet}^\top\log(\mu^Z) - \left[\mathbf{1}^\top\mu^X\right]\left[\mathbf{1}^\top\mu^Z\right]
\end{aligned}
\tag{B.1}
$$

S2.2 Model factorisation: NB model

Following the log-likelihood function in Equation (2.7),

$$
\begin{aligned}
l(\beta,\alpha) &= \sum_{i=1}^{M}\sum_{j=1}^{N}\left[\log\Gamma(Y_{ij}+\alpha^{-1}) - \log\Gamma(Y_{ij}+1) - \log\Gamma(\alpha^{-1}) + Y_{ij}\log(\alpha\mu_{ij}) - (Y_{ij}+\alpha^{-1})\log(1+\alpha\mu_{ij})\right] \\
&= \sum_{i=1}^{M}\sum_{j=1}^{N}\left[\{\sum_{k=0}^{Y_{ij}-1}\log(k+\alpha^{-1})\} - \log\Gamma(Y_{ij}+1) + Y_{ij}\log(\alpha\mu_{ij}) - (Y_{ij}+\alpha^{-1})\log(1+\alpha\mu_{ij})\right] \\
&= \sum_{i=1}^{M}\sum_{j=1}^{N}\left[\{\sum_{k=0}^{Y_{ij}-1}\log(k+\alpha^{-1})\} - \log\Gamma(Y_{ij}+1) + Y_{ij}\log(\alpha) + Y_{ij}\log(\mu_{ij}) - (Y_{ij}+\alpha^{-1})\log(1+\alpha\mu_{ij})\right] \\
&= \left(\sum_{i=1}^{M}\sum_{j=1}^{N} Y_{ij}\log(\alpha^{-1}) - \sum_{i=1}^{M}\sum_{j=1}^{N}\log(1)\right) + \left(\sum_{i=1}^{M}\sum_{j=1}^{N} Y_{ij}\right)\log(\alpha) \\
&+ \sum_{i=1}^{M}\sum_{j=1}^{N} Y_{ij}\left(\sum_{k=1}^{P} X_{jk}\beta_k + \sum_{s=1}^{R} Z_{is}\gamma_s\right) - \sum_{i=1}^{M}\sum_{j=1}^{N}(Y_{ij}+\alpha^{-1})\log(1+\alpha\mu_{ij})
\end{aligned}
\tag{B.2}
$$

Here, the last term $\sum_{j=1}^{N}(Y_{ij}+\alpha^{-1})\log(1+\alpha\mu_{ij})$, is impractical to simplify, therefore, we consider a moment matching method similar to that in Appendix S1.3, $Y_{\bullet,j} \sim \mathrm{NB}(r'_j, p'_j)$ where

$$r'_j = \alpha^{-1}\frac{(\sum_{i=1}^{M}\mu_{ij})^2}{\sum_{i=1}^{M}\mu_{ij}^2} = \alpha^{-1}\frac{(\mu_j^X\sum_{i=1}^{M}\mu_i^Z)^2}{\sum_{i=1}^{M}(\mu_j^X\mu_i^Z)^2} = \alpha^{-1}\frac{(\mu_j^X)^2(\sum_{i=1}^{M}\mu_i^Z)^2}{\sum_{i=1}^{M}(\mu_j^X\mu_i^Z)^2}$$

$$p'_j = \frac{\sum_{i=1}^{M}\mu_{ij}^2}{\alpha^{-1}\sum_{i=1}^{M}\mu_{ij}+\sum_{i=1}^{M}\mu_{ij}^2} = \frac{\sum_{i=1}^{M}(\mu_j^X\mu_i^Z)^2}{\alpha^{-1}\sum_{i=1}^{M}(\mu_j^X\mu_i^Z)+\sum_{i=1}^{M}(\mu_j^X\mu_i^Z)^2} = \frac{\sum_{i=1}^{M}(\mu_j^X\mu_i^Z)^2}{\alpha^{-1}\mu_j^X\sum_{i=1}^{M}\mu_i^Z+\sum_{i=1}^{M}(\mu_j^X\mu_i^Z)^2} \tag{B.3}$$

And the parameter α' of excess variance in the NB approximation is

$$\frac{1}{\alpha'} = \frac{1}{\alpha}\frac{(\sum_{i=1}^{M}\mu_{ij})^2}{\sum_{i=1}^{M}\mu_{ij}^2} \Rightarrow \alpha' = \frac{\sum_{i=1}^{M}\mu_{ij}^2}{(\sum_{i=1}^{M}\mu_{ij})^2}\alpha = \frac{\sum_{i=1}^{M}(\mu_j^X\mu_i^Z)^2}{(\mu_j^X)^2(\sum_{i=1}^{M}\mu_i^Z)^2}\alpha \tag{B.4}$$

S2.3 *Model factorisation: clustered NB model*

Following the total log-likelihood function in Appendix S1.6, we incorporate the effect of study-level covariates into the Clustered NB model,

$$\begin{aligned}
l(\beta,\alpha) &= Mv\log(v) - M\log\Gamma(v) + \sum_{i=1}^{M}\log\Gamma(Y_{i,\cdot}+v) - \sum_{i=1}^{M}(Y_{i,\cdot}+v)\log(v+\mu_{i,\cdot}) + \sum_{i=1}^{M}\sum_{j=1}^{N}Y_{ij}\log(\mu_{ij}) \\
&= Mv\log(v) - M\log\Gamma(v) + \sum_{g=1}^{B}\sum_{i\in I_g}\log\Gamma(Y_{i,\cdot}+v) - \sum_{g=1}^{B}\sum_{i\in I_g}(Y_{i,\cdot}+v)\log(v+\mu_{i,\cdot}) \\
&+ \sum_{g=1}^{B}\left(\sum_{j=1}^{N}\sum_{i\in I_g}Y_{ij}\right)\left(\sum_{k=1}^{P}X_{jk}\beta_{g(i)k} + \sum_{s=1}^{R}Z_{is}\gamma_s\right) \\
&= Mv\log(v) - M\log\Gamma(v) + \sum_{g=1}^{B}\sum_{i\in I_g}\log\Gamma(Y_{i,\cdot}+v) - \sum_{g=1}^{B}\sum_{i\in I_g}(Y_{i,\cdot}+v)\log(v+\mu_{i,\cdot}) \\
&+ \sum_{g=1}^{B}\left[\sum_{j=1}^{N}Y_{gj}\sum_{k=1}^{P}X_{jk}\beta_{g(i)k}\right] + \sum_{i=1}^{M}Y_{i,\cdot}\sum_{s=1}^{R}Z_{is}\gamma_s \\
&= Mv\log(v) - M\log\Gamma(v) + \sum_{g=1}^{B}\sum_{i\in I_g}\log\Gamma(Y_{i,\cdot}+v) - \sum_{g=1}^{B}\sum_{i\in I_g}(Y_{i,\cdot}+v)\log(v+\mu_{i,\cdot}) \\
&+ \sum_{g=1}^{B}Y_g^\top\log(\mu_g^X) + Y_{,\cdot}^\top\log(\mu^Z)
\end{aligned} \tag{B.5}$$

S2.4 *Using IRLS to Optimize the Quasi-Poisson Model*

Previously, we optimised the regression coefficients for likelihood-based models (e.g., Poisson, NB and clustered NB models) using Fisher scoring or L-BFGS algorithm. However, for Quasi-likelihood models (e.g., Quasi-Poisson model), where exact likelihood functions are computationally infeasible, we use the Iteratively Reweighted Least Squares (IRLS) method to iteratively determine the optimal regression coefficients.

An ordinary one-parameter exponential family of density functions can be written as,

$$f_\mu(y) = e^{\eta y-\psi(\mu)}\cdot[dG(y)] \tag{B.6}$$

Here μ is the expectation parameter, $\mu = \int_{-\infty}^{\infty} yf(y)dG(y)$; y is the natural statistic; η is the natural or canonical parameter, a monotone function of μ; $\psi(\mu)$ is a normalizing function, chosen to make the density integrate to 1. $G(y)$ is the *carrier measure* for the exponential family so that $Pr_\mu\{A\} = \int_A f_\mu(y)dG(y)$ for measurable sets A.

The Poisson model belongs to the exponential family, as its probability density function can be written as,

$$\begin{aligned} f_\mu(y) &= \frac{\mu^y}{y!}e^{-\mu} \\ &= \exp[y\log\mu - \mu - \log(y!)] \end{aligned} \tag{B.7}$$

where $\eta = \log(\mu)$ and $\psi(\mu) = \mu + \log(y!)$.

The double exponential families include an extra parameter θ to allow for over-dispersion, so that $Var(y) = \frac{\mathbb{E}(y)}{\theta}$. The probability distribution function can be written as,

$$\tilde{f}_{\mu,\theta}(y) = c(\mu.\theta)\theta^{\frac{1}{2}}\{f_\mu(y)\}^\theta\{f_y(y)\}^{1-\theta}[dG(y)] \tag{B.8}$$

The constant $c(\mu,\theta)$ is defined to make $\int_{-\infty}^{\infty}\tilde{f}_{\mu,\theta}(y)dG(y) = 1$. Therefore, the probability of Quasi-Poisson with unknown parameters μ and θ is written as,

$$\begin{aligned} \tilde{f}_{\mu,\theta}(y) &= c(\mu,\theta)\theta^{\frac{1}{2}}\left[\exp(y\log\mu - \mu - \log(y!))\right]^\theta\left[\exp(y\log y - y - \log(y!))\right]^{1-\theta} \\ &= c(\mu,\theta)\theta^{\frac{1}{2}}\exp\{\theta[y\log\mu - \mu - \log(y!)] + (1-\theta)[y\log y - y - \log(y!)]\} \end{aligned} \tag{B.9}$$

As a discrete distribution, we can choose a maximum count data n, compute the probability $\tilde{f}_{\mu,\theta}(y)$ of possible count data $y = 1, 2, \cdots, n$ and scale up the sum to 1.

The updating equation for the kth iteration of IRLS is given by,

$$\hat{\beta}^{[j+1]} = (X^TW^{[j]}X)^{-1}X^TW^{[j]}\xi^{[j]} \tag{B.10}$$

where $\xi^{[j]} = \eta^{[j]} + (W^{[j]})^{-1}(y - \mu^{[j]})$ and link function $\eta^{[k]} = g(\mu^{[k]}) = X\beta^{[k]}$. $W^{[j]}$ is a diagonal matrix with elements,

$$\frac{\left[\frac{\partial g^{-1}(\eta_1^{[j]})}{\partial\eta_1^{[j]}}\right]^2}{v(\mu_1^{[j]})}, \cdots, \frac{\left[\frac{\partial g^{-1}(\eta_n^{[j]})}{\partial\eta_n^{[j]}}\right]^2}{v(\mu_n^{[j]})} \tag{B.11}$$

For Quasi-Poisson model,

$$W = diag(\frac{\mu_1^2}{\theta\mu_1}, \cdots, \frac{\mu_n^2}{\theta\mu_n}) = diag(\frac{\mu_1}{\theta}, \cdots, \frac{\mu_n}{\theta}) \tag{B.12}$$

and equation B.10 can be written as,

$$\hat{\beta}^{[j+1]} = \beta^{[j]} + (X^\top W^{[j]} X)^{-1} X^\top (y - \mu^{[j]}) \tag{B.13}$$

S2.5 *Using the Delta Method to Estimate the Standard Errors of η^X and μ^X*

In the test of homogeneity to identify activation regions, the standard error for β (regression coefficients) can be asymptotically estimated from the inverse of the observed Fisher Information matrix. Additionally, the standard error of the linear response η_{ij}^X ($\eta^X = X\beta$), can be estimated using the delta method.

By definition, the optimal regression coefficients $\hat{\beta}$ converges in probability to its true value β, and a central limit theorem can be applied to obtain asymptotic normality,

$$\sqrt{n}(\beta - \hat{\beta}) \xrightarrow{D} N(0, \Sigma) \tag{B.14}$$

where n is the number of observations and Σ is a (symmetric positive semi-definite) covariance matrix.

$$\begin{aligned} Var(\hat{\eta}^X) &= Var(X\hat{\beta}) \\ &= XCov(\hat{\beta})X^\top = X\Sigma X^\top \end{aligned} \tag{B.15}$$

Since keeping only the first two terms of the Taylor series, and using vector notation for the

gradient, we can estimate μ^X as

$$\begin{aligned}
\hat{\mu}^X &= \exp(\hat{\eta}^X) = \exp(\eta^X) + \nabla \exp(\eta^X)(\beta - \hat{\beta}) \\
&= \exp(\eta^X) + \mathrm{diag}(\exp(\eta^X))(\eta^X - \hat{\eta}^X) \\
\mathrm{Var}(\mu^X) &= \mathrm{Var}\left(\exp(\eta^X) + \mathrm{diag}(\exp(\eta^X))(\eta^X - \hat{\eta}^X)\right) \\
&= \mathrm{Var}(\exp(\eta^X)) + \mathrm{Var}(\mathrm{diag}(\exp(\eta^X)) \cdot (\eta^X - \hat{\eta}^X)) \\
&= \mathrm{Var}(\exp(\eta^X)) + \mathrm{Var}(\mathrm{diag}(\exp(\eta^X)) \cdot \eta^X) - \mathrm{Var}(\mathrm{diag}(\exp(\eta^X)) \cdot \hat{\eta}^X) \\
&= \mathrm{Var}(\mathrm{diag}(\exp(\eta^X)) \cdot \hat{\eta}^X) \\
&= \mathrm{diag}(\exp(\eta^X))\,\mathrm{Var}(\hat{\eta}^X)\,\mathrm{diag}(\exp(\eta^X)) \\
&= \mathrm{diag}(\exp(\eta^X)) X\Sigma X^\top \mathrm{diag}(\exp(\eta^X))
\end{aligned} \tag{B.16}$$

The delta method therefore implies that

$$\sqrt{n}(\mu^X - \hat{\mu}^X) \xrightarrow{D} N\left[0, \mathrm{diag}(\exp(\eta^X)) X\Sigma X^\top \mathrm{diag}(\exp(\eta^X))\right]$$

S3. Simulation studies to validate the spatial design matrix in CBMR

S3.1 *Cubic B-spline basis and Gaussian kernel basis functions*

To rigorously evaluate the robustness of CBMR with a spatial B-spline basis matrix, we have conducted simulation experiments to demonstrate the effectiveness of the CBMR approach. These experiments are conducted in 2D settings, with either homogeneous intensity over the space or two bump signals located at the top-left and bottom-right corners of the image (these bump signals are constructed using Gaussian distributions, on the basis of background noise). The spatial design matrix is generated either with a cubic B-spline basis or a Gaussian kernel basis.

The specific setups are as follows:

- 2D simulation with homogeneous intensity: the intensity values are 0.01, 0.1 and 1.

Intensity Intensity	Spatial matrix	Bias($\hat{\mu}$)	Std($\hat{\mu}$)	MSE($\hat{\mu}$)	Rel. diff in ML
0.01	B-spline basis	6.6516×10^{-6}	2.1370×10^{-2}	4.5666×10^{-6}	0.0402%
0.01	Gaussian kernel	5.5857×10^{-6}	2.2222×10^{-2}	4.9388×10^{-6}	
0.1	B-spline basis	-5.2330×10^{-6}	6.7805×10^{-3}	4.5978×10^{-5}	−0.0007%
0.1	Gaussian kernel	-5.9101×10^{-6}	7.0259×10^{-3}	4.9366×10^{-5}	
1.0	B-spline basis	7.2501×10^{-6}	2.136×10^{-2}	4.5628×10^{-4}	−0.0002%
1.0	Gaussian kernel	8.2326×10^{-6}	2.2054×10^{-2}	4.8639×10^{-4}	

Table S5: Bias, Std, MSE and relative difference in maximised log-likelihood (ML) for homogeneous spatial intensity

- 2D simulation with bump signals: generated with two Gaussian distributions at the top-left and bottom-right corners of the image, combined with background noise. The expected numbers of foci per contrast are 2, 20, 200, respectively.

We then evaluate the mean, bias and mean square error (MSE) of the intensity estimates, denoted as $\hat{\mu}$ (averaged across space), as well as the relative difference in maximised log-likelihood (ML) between spatial design matrix generated with a B-spline basis or a Gaussian kernel, in each simulation scenario (see tables S5 and S6for details).

Therefore, we believe that we've substantiated the effectiveness of spatial design matrices created using either a cubic B-spline basis or a Gaussian kernel in the estimation of spatial intensity within the CBMR framework, and the difference between these two approaches is found to be minimal.

S3.2 *Sensitivity analysis on knots locations, numbers and degree of B-spline basis*

In all experiments described in Section 4, we have consistently used a cubic B-spline basis with a knot spacing of 10 voxels, equivalent to 20 mm. (Henceforth, we will refer knot spacing only using voxel units.) In this section we demonstrate that this choice is supported by evidence from

Expected n_foci	Spatial matrix	Bias($\hat{\mu}$)	Std($\hat{\mu}$)	MSE($\hat{\mu}$)	Rel.diff in ML
2	B-spline basis	$-6.1176e^{-6}$	$1.0586e^{-4}$	$1.1244e^{-8}$	1.0890%
2	Gaussian kernel	$-5.6949e^{-6}$	$9.1947e^{-5}$	$8.4867e^{-9}$	
20	B-spline basis	$-6.2624e^{-7}$	$2.4268e^{-4}$	5.88964^{-8}	0.08738%
20	Gaussian kernel	$-3.5496e^{-8}$	$2.5395e^{-4}$	$6.4492e^{-8}$	
200	B-spline basis	$-1.29598e^{-5}$	$5.9853e^{-4}$	$3.5840e^{-7}$	1.4398%
200	Gaussian kernel	$-1.1154e^{-5}$	$7.8658e^{-4}$	$6.1883e^{-7}$	

Table S6: Bias, Std, MSE and relative bias of difference in maximised log-likelihood values for inhomogeneous spatial intensity with two bump signals

experiments, not just prior knowledge or pragmatic considerations. We will explore how variations in the number of knots, their locations, and the degree of the B-spline basis affect the downstream meta-regression approach. This analysis will allow us to provide practical recommendations for parameter selection in future applications of the CBMR approach.

We perform sensitivity analysis using both simulated settings and real datasets. The specific setups are as follows:

- Simulation: We create an underlying intensity function, which is the sum of the following two components:
  - An intensity function that is the sum of two scaled two Gaussian probability density functions with centres at $(25, 25, 25)$ and $(65, 65, 65)$ in voxel space, with covariance matrix $5 \cdot I_3$ (i.e. standard deviation $\sqrt{5}$, FWHM ≈ 5.3). We used a scale factor of 5, which produced an average of 8.44 foci locations per study within the brain mask.
  - a spatial homogeneous background intensity of 10^{-6} across the entire brain image.

  Using this intensity function we simulated data at each voxel according to a Negative Binomial distribution, with inflation factor $\alpha = 0.5$.

This procedure is repeated 100 times to compare the relative mean, standard deviation (SD) and root mean squared error (RMSE) of the estimated intensity for the following experiment settings. Additionally, we evaluate the relative difference in maximised log-likelihood values.

We consider the following variations:

  - Baseline experiment: Spatial design matrix uses a cubic B-spline basis with knots spacing of 10 voxels (as in body of the paper);
  - Quadratic vs Cubic B-spline basis: Design matrix uses a quadratic B-spline bases, while keeping the knot configurations from the baseline experiment.
  - Half interval shift: To understand the impact of knot locations, we shift all knot locations by half an interval to the right, keeping the other knot and spline configurations unchanged from the baseline experiment.
  - Knot spacing: We vary knot spacing (from 4 voxels to 40 voxels) and analyse a data sufficiency index (described below) to provide practical recommendations for the future application of the CBMR approach.

- Real datasets: We run CBMR with a Negative Binomial (NB) model on 20 cognitive datasets (details provided in Table 1) across various knot spacings (from 4 voxels to 40 voxels). We use the CBMR-estimated intensity with a knot spacing of 10 voxels as the baseline experiment, and compare the relative bias, standard deviation, difference in maximised log-likelihood values and RMSE of estimated intensity functions generated by other knot spacings.

For simulated data, since the actual underlying intensity function is known, we compare the CBMR-estimated intensities across various experiment settings with the actual underlying in-

B-spline basis	1/2 int. shift	Rel.bias($\hat{\mu}$)	Rel.std($\hat{\mu}$)	RMSE($\hat{\mu}$)	Rel. diff in ML
**Cubic**	**No**	4.3941%	13.8096%	$5.6967e^{-6}$	-2.6157%
Quadratic	No	4.1127%	12.2380%	$5.3711e^{-6}$	-2.5617%
Cubic	Yes	4.7631%	12.7943%	$5.9858e^{-6}$	-2.4767%
Quadratic	Yes	4.4988%	14.2543%	$5.3105e^{-6}$	-2.5986%

Table S7: Relative Mean, relative SD, RMSE and relative bias of difference in maximised log-likelihood values for the simulated dataset, with spline spacing of 5 voxels. The baseline experiment results are highlighted in bold.

tensity function. The results are summarised in Table S7. Our findings indicate that both cubic and quadratic B-spline bases, regardless of whether the knot locations are original or shifted to the right by half an interval, provide comparable levels of relative bias (ranging between 4.1127% and 4.7631%), relative standard deviation (ranging between 12.2380% and 14.2543%) and similar level of RMSE (ranging between 5.3105×10^{-6} and 5.9858×10^{-6}). (We examined the spatial variation in bias, and found it generally occurred in background area where a simulated dataset had no foci and our method estimated a negligible intensity, below the true 10^{-6} background intensity.) We also compared the maximised log-likelihood values to those evaluated with true μ values. This comparison also shows small variations. These findings indicate that the knot locations and the degree of the B-spline basis are not significant factor influencing the estimated intensity function of the CBMR model.

Additionally, to provide practical guidelines for parameter selection in future applications of the CBMR approach, we introduce a data sufficiency index. This index will help to identify the minimum foci contributions per basis element required to ensure the effective functioning of the CBMR method. Since our basis is a partition of unity, when we project the foci onto the basis functions and sum over voxels, the total foci count is $\sum_{jk} X_{jk} Y_{\bullet,j}$, recalling that $Y_{\bullet,j}$ is sum count over studies at voxel j and X is the $(N \times P)$ is the spatial design matrix. Thus we can consider

B-spline basis	knot spacing	Rel.bias($\hat{\mu}$)	Rel.SD($\hat{\mu}$)	RMSE($\hat{\mu}$)	Rel.diff in ML
Cubic	4	4.3364%	6.8069%	$5.9917e^{-6}$	−3.0709%
**Cubic**	**5**	4.3941%	13.8096%	$5.6967e^{-6}$	−2.6157%
Cubic	7.5	3.2286%	10.2068%	$3.9609e^{-6}$	−0.9849%
Cubic	10	1.2322%	10.7362%	$3.4287e^{-6}$	−0.3988%
Cubic	15	−0.6630%	4.7229%	$3.7970e^{-6}$	0.1300%
Cubic	20	−4.7553%	5.6380%	$5.1880e^{-6}$	0.5380%
Cubic	30	−10.0698%	7.0024%	$9.1770e^{-6}$	2.4265%
Cubic	40	−13.5894%	4.4585%	$1.0088e^{-5}$	2.77314%

Table S8: Relative mean, SD, RMSE and relative bias of difference in maximised log-likelihood values of CBMR results across various knot spacings of cubic B-spline bases in the simulated dataset. The baseline experiment results are highlighted in bold.

B-spline basis	knot spacing	1/2 interval shift	Max total foci contribution
Cubic	4.0	No	13.7408
**Cubic**	5.0	**No**	18.7957
Cubic	7.5	No	45.3228
Cubic	10.0	No	73.8215
Cubic	15.0	No	182.0279
Cubic	20.0	No	286.8437
Cubic	30.0	No	375.7031
Cubic	40.0	No	362.0541

Table S9: Maximum of total foci contribution per basis element for the Cue Reactivity dataset, with the baseline experiment in bold.

the total contribution to each basis element in this simplified setting by

$$\left[\sum_j X_{j1} Y_{\bullet,j}, \sum_j X_{j2} Y_{\bullet,j}, \dots, \sum_j X_{jP} Y_{\bullet,j}\right],$$

and summarise this P-vector by the maximum total foci contribution to any one basis element

$$\max_{k=1,\dots,P} \sum_j X_{jk} Y_{\bullet,j}.$$

Using the maximum total foci contribution per basis element as the data sufficiency index, we calculated and compared this index across various knot spacings for one specific dataset, the Cue Reactivity, in Table S9. This analysis reveals that the maximum total foci contribution per basis

element increases almost monotonically with wider spline spacings of the B-spline basis, reaching maximum levels at a spacing of 30 voxels, beyond which no significant increases were observed. Additionally, we compared the relative bias, standard deviation (SD), difference in maximised log-likelihood values and RMSE in Table S9. It indicates that all cubic B-spline basis functions with knot spacings between 4 and 20 voxels are capable of producing very accurate estimations of the intensity function. Among these, a spline spacing of 15 voxels is the best option, giving rise to the lowest relative bias, standard deviation, difference in maximised likelihood value and RMSE. Previously, our practical experience with the CBMR model indicated that a spline spacing of 10 voxels in datasets with low foci counts can lead to singularity issues in Fisher information and result in inaccurate standard errors. As a result, here we explore how to determine the optimal spline spacing for datasets of different sizes to avoid numerical singularity issues as guided by our data sufficiency index.

In table S12, we used the intensity estimation from a spline spacing of 10 voxels as the reference (since the true underlying intensity function is unknown) and compared the relative bias, standard deviation, and RMSE of the CBMR with the NB model across various spline spacings between 4 and 40. We observed that the dissimilarity (in terms of relative bias and RMSE) increases as the spline spacing diverges from the reference with spline spacing of 10 voxels, while the relative standard deviation decreases as the spline spacing widens. This indicates that spline spacing significantly influences intensity estimation in real datasets, with larger spline spacings giving rise to less variation in intensity estimation across different voxel locations. Additionally, Figure S8 and Figure S9, present the results from 100 experiments conducted the CBMR with the NB model for each of the 20 cognitive datasets (details provided in Table 1) where these datasets were categorised into four groups according to their total foci counts. We calculated the rate of convergence failures over 100 realisations and compared these rates across various spline spacings and computed data sufficiency indiex. (Each optimisation starts with random sampling of β's;

here we only use one initialisation for each realisation, though in practice we re-initialise on convergence failure for real data analyses).

Our analysis revealed that different groups of real datasets require different spline spacings (or data sufficiency indices), as outlined below:

- For datasets with fewer than 500 foci counts, we recommend a spline spacing of 30 voxels or a data sufficiency index greater than 20, as larger spline spacings are associated with a higher data sufficiency index which helps to avoid non-convergence.

- For datasets with foci counts ranging from 500 and 1500, a spline spacing of 20 voxels or a data sufficiency index greater than 20 is recommended. At this spacing, the rates of failure to converge have been controlled at a very low level, and no further reductions in failure rates were observed with larger spline spacings.

- For datasets containing between 1500 and 4000 foci, we recommend a spline spacing of 15 voxels or a data sufficiecy index more than 65. At this level, the rate of failures decreased to zero, indicating successful convergence in all 100 experiments.

- For datasets with foci counts more than 4000, a spline spacing of 10 voxels or a data sufficiency index more than 65 is recommended. For these large datasets, there is sufficient foci contribution per basis element even with larger spline spacing, and a spline spacing of 10 voxels helps avoid numerical singularity in Fisher information.

We have also compared the maximised log-likelihood for each of the 20 cognitive datasets (details provided in Table 1) across 100 runs of the CBMR with the NB model, using different spacings of cubic B-spline bases and a reference of spline spacing of 10 voxels. As depicted in Figure S10, maximised log-likelihood values decrease with larger B-spline basis knot spacings. These

Dataset		knot spacing							
		4	5	7.5	10	15	20	30	40
1	Bias	85.87%	65.5%	38.48%	0.0%	31.83%	44.53%	52.55%	55.04
	SD	176.33%	137.61%	104.13%	87.33%	75.78%	63.78%	61.67%	54.83%
	RMSE	$7.0767e^{-5}$	$5.4990e^{-5}$	$4.0051e^{-5}$	$3.1499e^{-5}$	$2.9655e^{-5}$	$2.8073e^{-5}$	$2.9245e^{-5}$	$2.8045e^{-5}$
2	Bias	191.6%	188.56%	140.02%	0.0%	126.30%	143.2%	153.68%	159.95
	SD	3868.61%	3681.34%	1894.89%	728.63%	224.51%	171.22%	122.16%	124.82%
	RMSE	$1.1887e^{-3}$	$1.1313e^{-3}$	$5.8314e^{-4}$	$2.2362e^{-4}$	$7.9070e^{-5}$	$6.8522e^{-5}$	$6.0274e^{-5}$	$6.2290e^{-5}$
3	Bias	191.42%	160.58%	74.21%	0.0%	55.01%	68.62%	75.34%	82.44
	SD	1582.41%	920.24%	211.92%	130.73%	96.93%	78.91%	69.44%	75.32%
	RMSE	$5.2983e^{-4}$	$3.1051e^{-4}$	$7.4638e^{-5}$	$4.3454e^{-5}$	$3.7049e^{-5}$	$3.4764e^{-5}$	$3.4059e^{-5}$	$3.7122e^{-5}$
4	Bias	166.95%	130.33%	63.06%	0.0%	43.94%	50.54%	57.60%	60.04
	SD	896.18%	461.70%	166.37%	116.22%	87.21%	78.23%	70.80%	67.83%
	RMSE	$1.7039e{-3}$	$8.9665e^{-4}$	$3.3253e^{-4}$	$2.1724e^{-4}$	$1.8251e^{-4}$	$1.7404e^{-4}$	$1.7054e^{-4}$	$1.6925e^{-4}$
5	Bias	102.27%	77.41%	41.89%	0.0%	36.68%	48.8%	57.64%	61.57
	SD	264.64%	190.12%	136.72%	115.62%	98.71%	86.79%	80.33%	75.94%
	RMSE	$1.4387e^{-4}$	$1.0409e^{-4}$	$7.2508e^{-5}$	$5.8621e^{-5}$	$5.3398e^{-5}$	$5.0497e^{-5}$	$5.0148e^{-5}$	$4.9595e^{-5}$
6	Bias	98.49%	71.38%	39.93%	0.0%	33.78%	44.92%	51.31%	54.61
	SD	228.12%	161.59%	118.08%	99.14%	83.03%	71.05%	67.46%	59.95
	RMSE	$1.1348e^{-4}$	$8.0678e^{-5}$	$5.6927e^{-5}$	$4.5275e^{-5}$	$4.0936e^{-5}$	$3.8389e^{-5}$	$3.8707e^{-5}$	$3.7036e^{-5}$
7	Bias	154.98%	116.96%	60.53%	0.0%	43.95%	58.60%	72.66%	76.76
	SD	730.71%	381.54%	190.41%	137.53%	115.32%	94.82%	81.46%	71.76
	RMSE	$2.7209e^{-4}$	$1.4536e^{-4}$	$7.2779e^{-5}$	$5.0098e^{-5}$	$4.4953e^{-5}$	$4.0603e^{-5}$	$3.9760e^{-5}$	$3.8275e^{-5}$
8	Bias	80.82%	61.42%	38.33%	0.0%	32.18%	41.22%	50.46%	53.56
	SD	196.23%	163.07%	135.44%	109.80%	96.08%	88.78%	81.64%	79.0
	RMSE	$7.3694e^{-5}$	$6.0508e^{-5}$	$4.8879e^{-5}$	$3.8129e^{-5}$	$3.5185e^{-5}$	$3.3990e^{-5}$	$3.3329e6{-5}$	$3.3143e^{-5}$
9	Bias	197.88%	185.54%	93.98%	0.0%	64.04%	75.64%	83.72%	85.85
	SD	2189.71%	1484.66%	290.07%	143.11%	91.46%	71.98%	63.13%	53.846
	RMSE	$9.9500e^{-4}$	$6.7712e^{-4}$	$1.3799e^{-4}$	$6.4767e^{-5}$	$5.0529e^{-5}$	$4.7252e^{-5}$	$4.7453e^{-5}$	$4.5860e^{-5}$
10	Bias	175.07%	129.23%	61.81%	0.0%	47.85%	57.55%	65.39%	64.84
	SD	924.02%	413.72%	149.45%	103.79%	74.75%	63.66%	60.01%	50.78
	RMSE	$3.8582e^{-4}$	$1.7781e^{-4}$	$6.6347e^{-5}$	$4.2580e^{-5}$	$3.6412e^{-5}$	$3.5208e^{-5}$	$3.6410e^{-5}$	$3.3786e^{-5}$
11	Bias	100.42%	74.67%	41.29%	0.0%	36.24%	51.44%	67.23%	74.56
	SD	302.45%	221.58%	167.48%	145.78%	130.8%	111.18%	95.27%	78.36
	RMSE	$1.5210e^{-4}$	$1.1160e^{-4}$	$8.2328e^{-5}$	$6.9579e^{-5}$	$6.4781e^{-5}$	$5.8466e^{-5}$	$5.5654e^{-5}$	$5.1627e^{-5}$
12	Bias	59.51%	46.86%	31.61%	0.0%	25.32%	34.54%	43.39%	46.43
	SD	133.43%	118.22%	102.98%	86.22%	78.66%	71.56%	66.92%	62.73
	RMSE	$8.1195e^{-5}$	$7.0674e^{-5}$	$5.9869e^{-5}$	$4.7917e^{-5}$	$4.5925e^{-5}$	$4.4162e6{-5}$	$4.4325e^{-5}$	$4.3373e^{-5}$
13	Bias	198.52%	192.38%	121.30%	0.0%	80.0%	91.32%	98.83%	99.61
	SD	2693.68%	2340.74%	585.23%	216.71%	116.21%	89.57%	78.86%	71.72
	RMSE	$4.5113e^{-4}$	$3.9228e^{-4}$	$9.9824e^{-5}$	$3.6196e^{-5}$	$2.3564e^{-5}$	$2.1364e^{-5}$	$2.1117e^{-5}$	$2.0501e^{-5}$
14	Bias	147.03%	147.06%	141.87%	0.0%	177.53%	185.28%	189.74%	192.12
	SD	5499.95%	5500.63%	5164.26%	2567.68%	382.94%	201.42%	129.23%	148.33
	RMSE	$1.4306e^{-3}$	$1.4307e^{-3}$	$1.3433e^{-3}$	$6.6762e^{-4}$	$1.0975e^{-4}$	$7.1158e^{-5}$	$5.9691e^{-5}$	$6.3110e^{-5}$
15	Bias	61.14%	48.01%	30.33%	0.0%	25.52%	36.86%	45.54%	50.12
	SD	135.25%	120.18%	105.26%	95.58%	87.24%	78.39%	73.93%	66.47
	RMSE	$7.7930e^{-5}$	$6.7951e^{-5}$	$5.7513e^{-5}$	$5.0183e^{-5}$	$4.7728e^{-5}$	$4.5481e^{-5}$	$4.5590e^{-5}$	$4.3711e^{-5}$
16	Bias	106.78%	79.12%	43.33%	0.0%	35.58%	50.5%	62.55%	65.18
	SD	292.45%	200.44%	143.39%	117.93%	101.4%	86.01%	78.87%	68.37
	RMSE	$1.0351e^{-4}$	$7.1642e^{-5}$	$4.9800e^{-5}$	$3.9206e^{-5}$	$3.5726e^{-5}$	$3.3160e^{-5}$	$3.3467e^{-5}$	$3.1405e^{-5}$

17	Bias	198.0%	193.39%	115.88%	0.0%	74.11%	85.73%	95.32%	98.57
	SD	2549.71%	2239.09%	471.09%	172.02%	96.78%	76.27%	63.3%	58.98
	RMSE	$5.1769e^{-4}$	$4.5495e^{-4}$	$9.8206e^{-5}$	$3.4822e^{-5}$	$2.4675e^{-5}$	$2.3228e^{-5}$	$2.3163e^{-5}$	$2.3253e^{-5}$
18	Bias	104.83%	77.16%	43.33%	0.0%	38.09%	59.29%	72.81%	78.34
	SD	340.22%	234.88%	187.97%	163.09%	145.22%	119.39%	106.13%	93.61
	RMSE	$1.6885e^{-4}$	$1.1726e^{-4}$	$9.1489e^{-5}$	$7.7354e^{-5}$	$7.1205e^{-5}$	$6.3224e^{-5}$	$6.1041e^{-5}$	$5.7894e^{-5}$
19	Bias	183.14%	169.13%	108.53%	0.0%	91.59%	108.5%	126.61%	130.17
	SD	3166.52%	2614.67%	1225.12%	610.3%	356.0%	283.14%	231.17%	207.65
	RMSE	$1.0340e^{-3}$	$8.5412e^{-4}$	$4.0093e^{-4}$	$1.9895e^{-4}$	$1.1983e^{-4}$	$9.8843e^{-5}$	$8.5919e^{-5}$	$7.9892e^{-5}$
20	Bias	163.83%	138.05%	73.84%	0.0%	62.3%	87.26%	96.93%	109.48
	SD	1318.59%	935.59%	345.83%	253.51%	196.2%	152.56%	138.69%	110.45
	$1.2851e^{-3}$	$9.1470e^{-4}$	$3.4203e^{-4}$	$2.4520e^{-4}$	$1.9910e^{-4}$	$1.6999e^{-4}$	$1.6366e^{-4}$	$1.5041e^{-4}$	

Table S12: Relative bias, standard deviation (SD) and RMSE for each of the 20 cognitive datasets (details provided in Table 1), with a spline spacing of 10 voxels as reference (as the underlying intensity function is unknown).

findings supports the conclusion that B-spline bases with smaller knot spacings are able to capture finer details and can produce more accurate estimations of intensity function. Although higher maximised log-likelihood values are associated with smaller spline spacing (less than the reference of 10 voxels), they mostly occur in datasets with relatively small foci counts. This could be linked to numerical singularity in estimating Fisher Information matrix and reduced accuracy in standard error estimates. Additionally, for any specific dataset, the relatively high maximised log-likelihood values associated with smaller spline knot spacings might due to overfitting.

S4. Statistical inference and generalised linear hypothesis testing

S4.1 *Contrasts on regression coefficient of study-level covariates*

To investigate the effects of study-level covariates (e.g., sample size, year of publication) on activation intensity estimation, we conduct generalised linear hypothesis testing on the regression coefficients γ. For every study-level covariate $\gamma_r, \forall r = 1, \cdots, s$,

- $H_0 : C_\gamma \gamma = C_\gamma[\gamma_1, \gamma_2, \cdots, \gamma_s]^T = \mathbf{0}^T_{m\times 1}$ where C_γ is the contrast matrix of size $m \times s (m \leqslant s)$

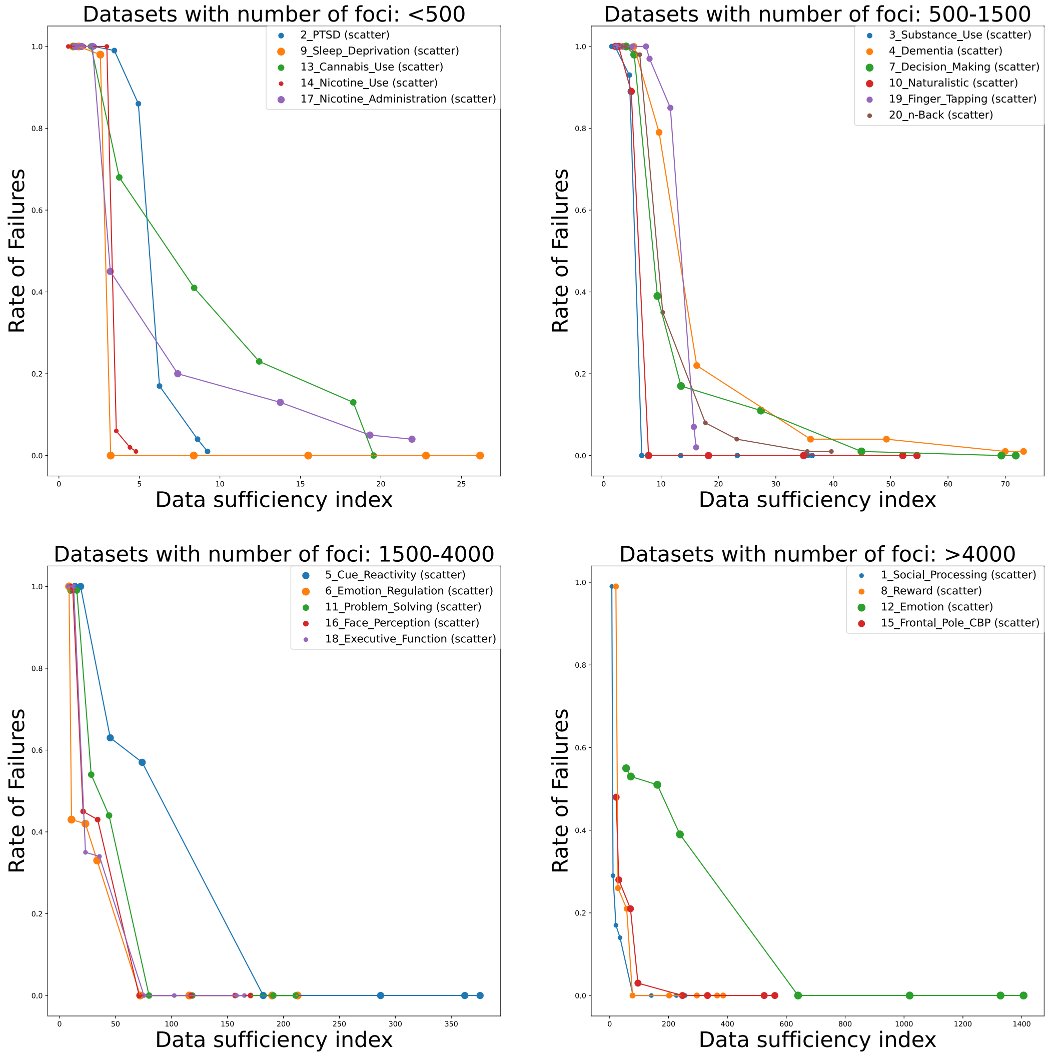

Figure S8: Rate of failure to converge in 100 experiments for each of the 20 datasets (details provided in Table 1) across various data sufficiency index, categorised into 4 groups according to their total foci counts.

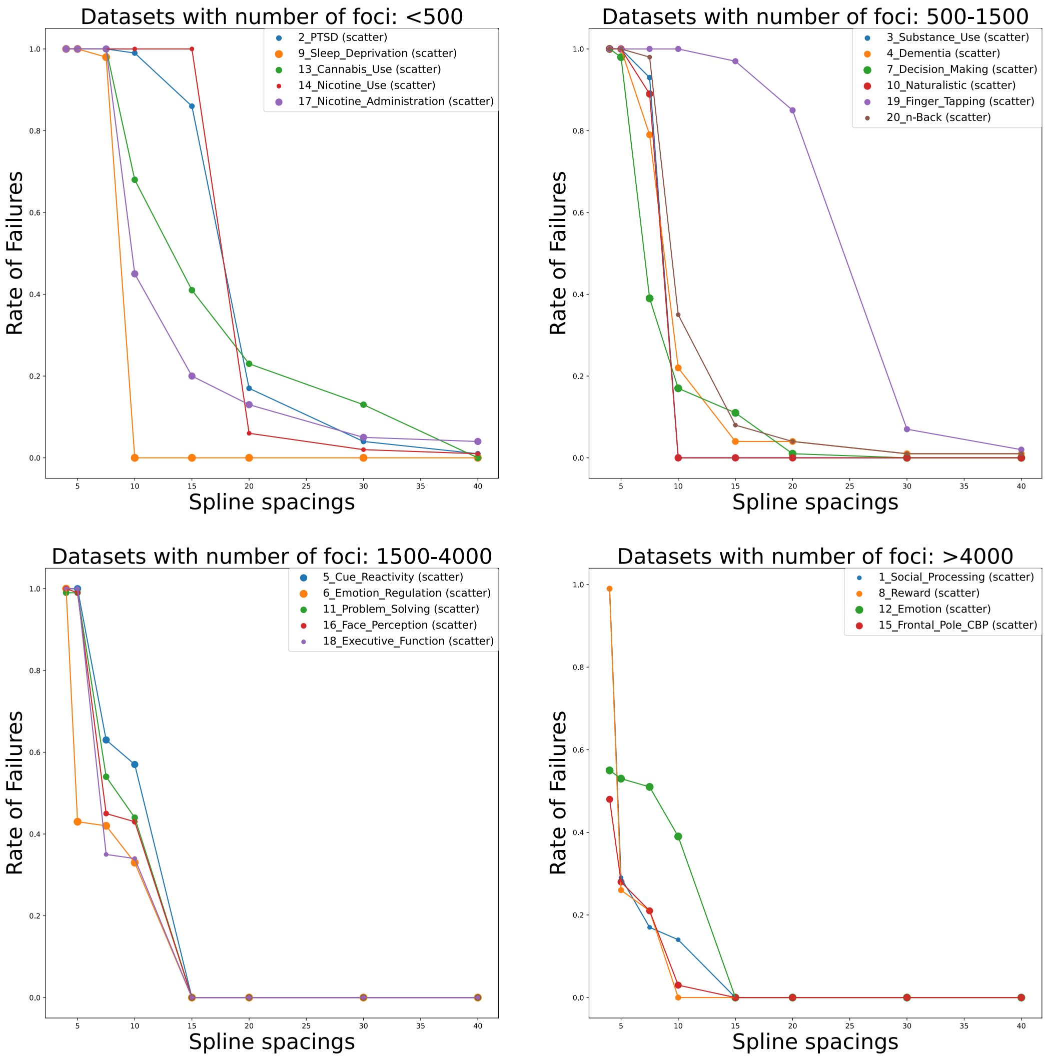

Figure S9: Rate of failure to converge in 100 experiments for each of the 20 datasets (details provided in Table 1) across various spline spacings, categorised into 4 groups according to their total foci counts.

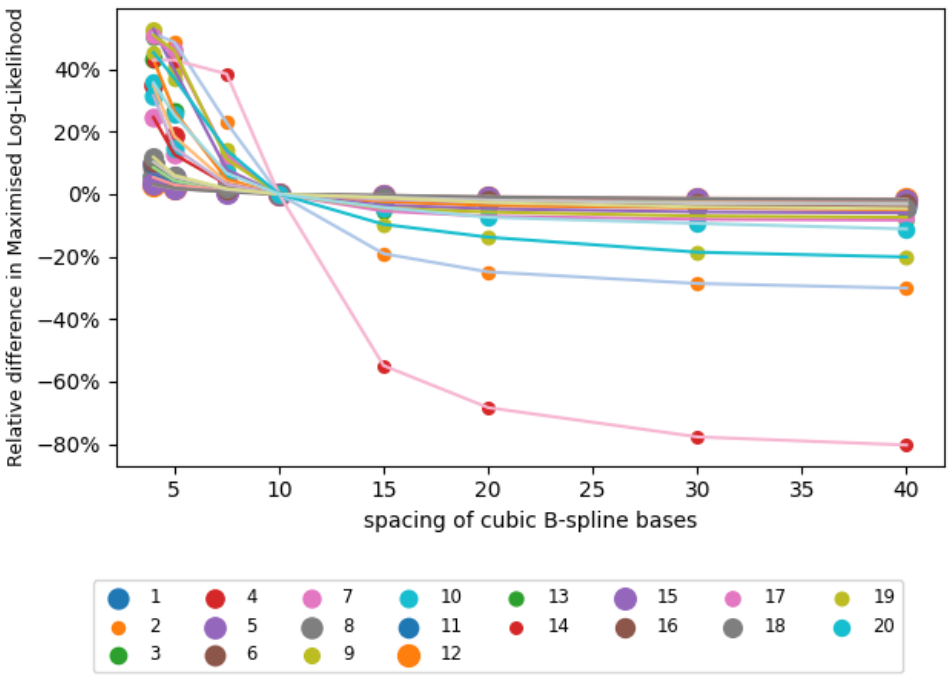

Figure S10: Relative difference in maximised log-likelihood for each of the 20 cognitive datasets (details provided in Table 1), with a spline spacing of 10 voxels as the reference. The size of scatters reflect the total foci counts for each dataset.

- $H_1 : C_\gamma \gamma = C_\gamma [\gamma_1, \gamma_2, \cdots, \gamma_s]^T \neq \mathbf{0}^T_{m\times 1}$

The covariance of the regression coefficient γ, $\text{Cov}_\gamma = \text{Cov}([\gamma_1, \gamma_2, \cdots, \gamma_s]^T)$ is approximated from the inverse of the Fisher Information matrix. According to the asymptotic normality of the maximum likelihood estimator,

$$\hat{\gamma} - \gamma \xrightarrow{D} N\left(\mathbf{0}^T_{s\times 1}, \text{Cov}_\gamma\right)$$

$$C_\gamma(\hat{\gamma} - \gamma) \xrightarrow{D} N\left(\mathbf{0}^T_{s\times 1}, C_\gamma \text{Cov}_\gamma C_\gamma^T\right)$$

$$C_\gamma \hat{\gamma} \xrightarrow{D} N\left(C_\gamma \gamma, C_\gamma \text{Cov}_\gamma C_\gamma^T\right)$$

Since a quadratic form of normal distribution has a Chi-square distribution,

$$(C_\gamma \hat{\gamma})^T (C_\gamma \text{Cov}_\gamma C_\gamma^T)^{-1} (C_\gamma \hat{\gamma}) \xrightarrow{D} \chi^2_m$$

for example, the contrast matrix $C_\gamma = [1, 0]$ or $[0, 1]$ is for testing if the regression coefficient of the 1st or 2nd study-level covariate is zero.

S4.2 *PP-plots of spatial homogeneity tests for each* 20 *meta-analytic datasets*

Previously, we displayed only the PP-plots of spatial homogeneity tests for four representative datasets in Section 4.1. Here, we will include all PP-plots for 20 meta-analytic datasets in Figure S11.

S4.3 *Likelihood-based comparison between Poisson, NB and clustered NB model*

To demonstrate the likelihood-based comparison between the Poisson, NB and clustered NB model, we plot the maximised log-likelihood and AIC for each of the 20 meta-analytic datasets in Figure S12 and Figure S13. We also conduct a Likelihood ratio test (LRT) to evaluate the trade-off between model sufficiency and complexity. Here, we only list the p-values of the LRT between the Poisson and clustered NB model in Table S13, as $p < 10^{-8}$ for the LRT between the Poisson and NB model for each of the 20 meta-analytic datasets.

Table S13: p-values of Likelihood Ratio test between Poisson and clustered NB model

Dataset	p-value	Dataset	p-value	Dataset	p-value
Social Processing	$p < 10^{-8}$	PTSD	$p < 10^{-8}$	Substance Use	$p < 10^{-8}$
Dementia	$p < 10^{-8}$	Cue Reactivity	$p < 10^{-8}$	Emotion Regulation	$p < 10^{-8}$
Decision Making	$p < 10^{-8}$	Reward	$p < 10^{-8}$	Sleep Deprivation	$p < 10^{-8}$
Naturalistic	$p < 10^{-8}$	Problem Solving	$p < 10^{-8}$	Emotion	$p < 10^{-8}$
Cannabis Use	1	Nicotine Use	$p < 10^{-8}$	Frontal Pole CBP	$p < 10^{-8}$
Face Perception	$p < 10^{-8}$	Nicotine Administration	0.99	Executive Function	$p < 10^{-8}$
Finger Tapping	0.99	n-Back	$p < 10^{-8}$		

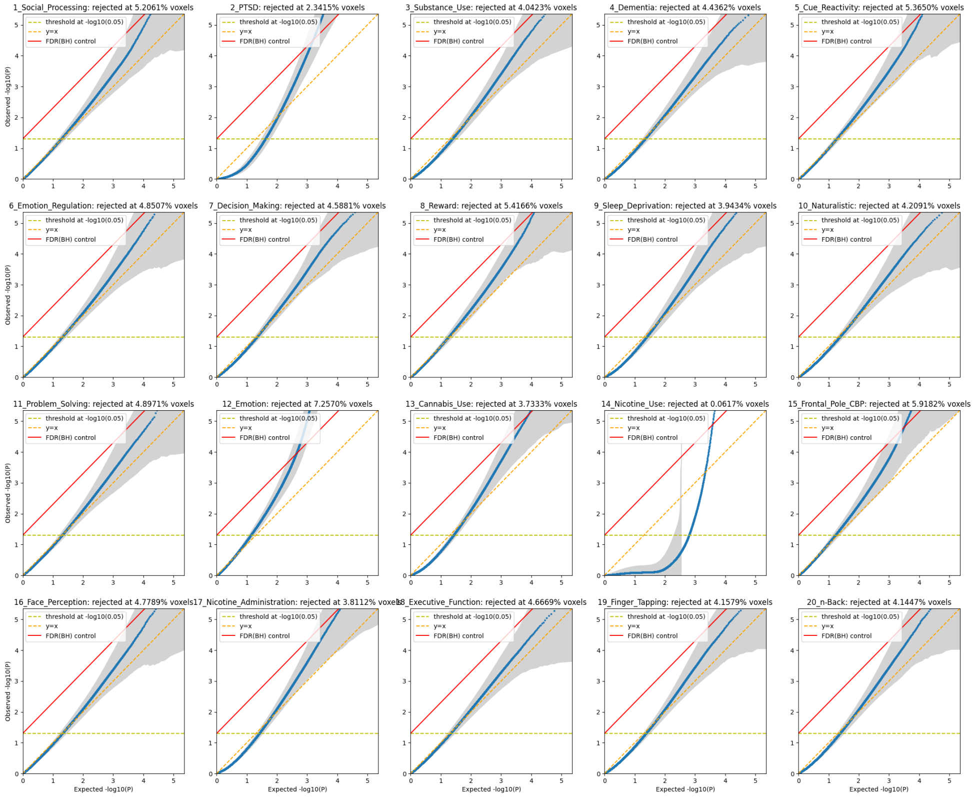

Figure S11: P-P plot of p-value (under $-\log_{10}$ scale) with all of 20 meta-analytic datasets, estimated by CBMR with NB model without study-level covariates, sampled with model-based approach.

S4.4 *Effect of study-level covariates*

Here, we investigate the effect of study-wise (square root) sample size and year of publication (after centring and standardisation) on each of the 20 meta-analytic datasets. Under the null hypothesis that regression coefficient of each covariate is not distinguishable from 0 ($\gamma_i = 0$ for $i = 1, 2$), we conduct hypothesis testing and summarise the $Z-$score and $p-$value in Table S14.

Table S14: Hypothesis testing on the effect of two study-level covariates on 20 meta-analytic datasets

	(Square root) sample size		Year of publication	
Dataset	$Z-$score	p-value	$Z-$score	p-value
Social Processing	10.9053	$p < 10^{-8}$	0.4164	0.6771
PTSD	2.8789	0.004	0.6029	0.5466
Substance Use	4.3887	1.1404×10^{-5}	6.8398	$p < 10^{-8}$
Dementia	20.7177	$p < 10^{-8}$	-1.3985	0.1620
Cue Reactivity	6.1454	$p < 10^{-8}$	-0.6880	0.4915
Emotion Regulation	6.8934	$p < 10^{-8}$	-3.9588	7.5329×10^{-5}
Decision Making	4.1104	3.9499×10^{-5}	0.1060	0.9156
Reward	-0.1228	0.9022	-	-
Sleep Deprivation	12.8765	$p < 10^{-8}$	0.4201	0.6744
Naturalistic	1.7038	0.0884	0.5395	0.5896
Problem Solving	4.3079	1.6485×10^{-5}	2.2789	0.0227
Cannabis Use	3.5915	3.2878×10^{-4}	2.2117	0.0270
Nicotine Use	5.0024	5.6631×10^{-7}	3.1836	0.0015
Frontal Pole CBP	5.5190	3.4101×10^{-8}	7.4040	$p < 10^{-8}$
Face Perception	3.4090	6.5212×10^{-4}	5.1018	3.3651×10^{-7}
Nicotine Administration	1.4594	0.1445	-1.0516	0.2930
Executive Function	1.6989	0.0932	0.5047	0.6138
Finger Tapping	-	-	0.1764	0.8600
n-Back	1.4616	0.1439	0.1239	0.9014

S5. Comparison with Bayesian LGCP regression

To validate the accuracy of intensity estimation and the detected activation regions generated by our CBMR approach, we reached out to the authors of the Bayesian log-Gaussian Cox Process regression (LGCP; as detailed in Samartsidis *and others*, 2019). LGCP is a fully Bayesian random-effect meta-regression model capable of estimating activation intensity through a simulation-based approximation of the posterior using Markov Chain Monte Carlo (MCMC) methods, and it also accounts for study-wise heterogeneity, similar to our CBMR approach. After obtaining the source code for their method, we applied it to the Cue Reactivity dataset for comparative analysis.

In the absence of p-value maps in the Bayesian LGCP approach, we chose to compare the estimated intensity maps generated by both the CBMR and LGCP methods, as presented in Figure S14a and figure S14b below (both are thresholded at 2×10^{-5}). Our analysis indicates significant consistency in the activation regions identified by these two approaches, particularly in the left cerebral cortex, frontal orbital cortex, insular cortex, and left and right accumbens. While we no-

ticed that the activation regions identified in the CBMR intensity map appeared more isolated. However, these regions appeared more cohesive in the p-value maps when we controlled the false discovery rate (FDR) using the BH method (see Figure 6).

Although the LGCP model is a robust Bayesian meta-regression model that includes random-effect terms to address study-wise heterogeneity, it's mathematically complex, and its MCMC algorithm requires approximately 30 hours of computational time on an NVIDIA Tesla K20c GPU card, in contrast to approximately 537.52 seconds (approx 9 minutes) required for the CBMR with the NB model (tested on an Intel Xeon Gold 6340R CPU) for the Cue Reactivity dataset. Therefore, we believe our CBMR approach offers a computationally efficient alternative to the LGCP model, while still achieving comparable accuracy.

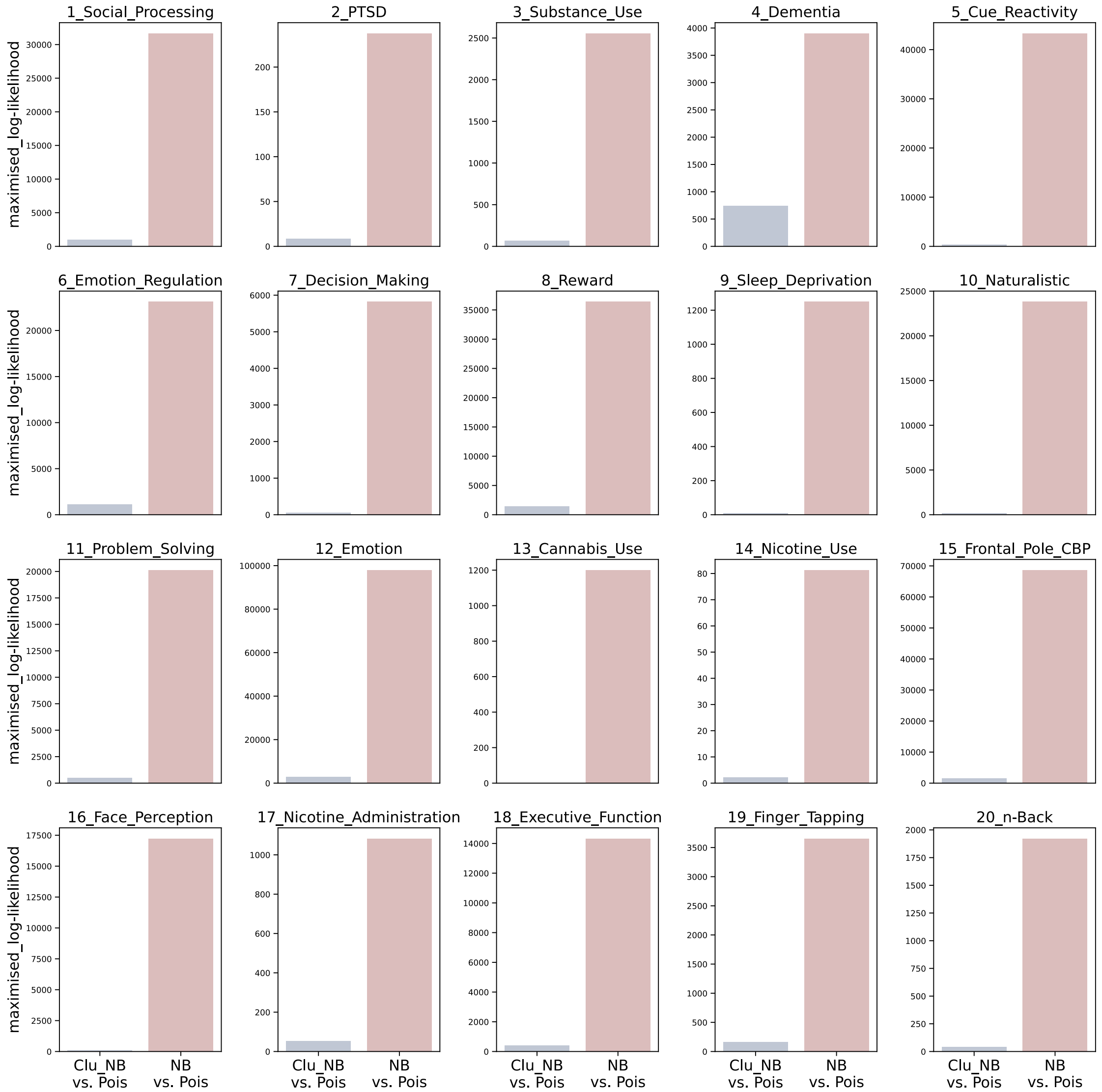

Figure S12: Likelihood-based comparison of CBMR with Poisson, NB and clustered NB models (difference in maximised log-likelihood values, with Poisson model as the reference). We found that the maximised log-likelihood value of NB model is always the highest, while for some datasets, the difference of maximised log-likelihood values between clustered NB and Poisson model is negligible, therefore, the existence of excess variance has been justified among CBMA data.

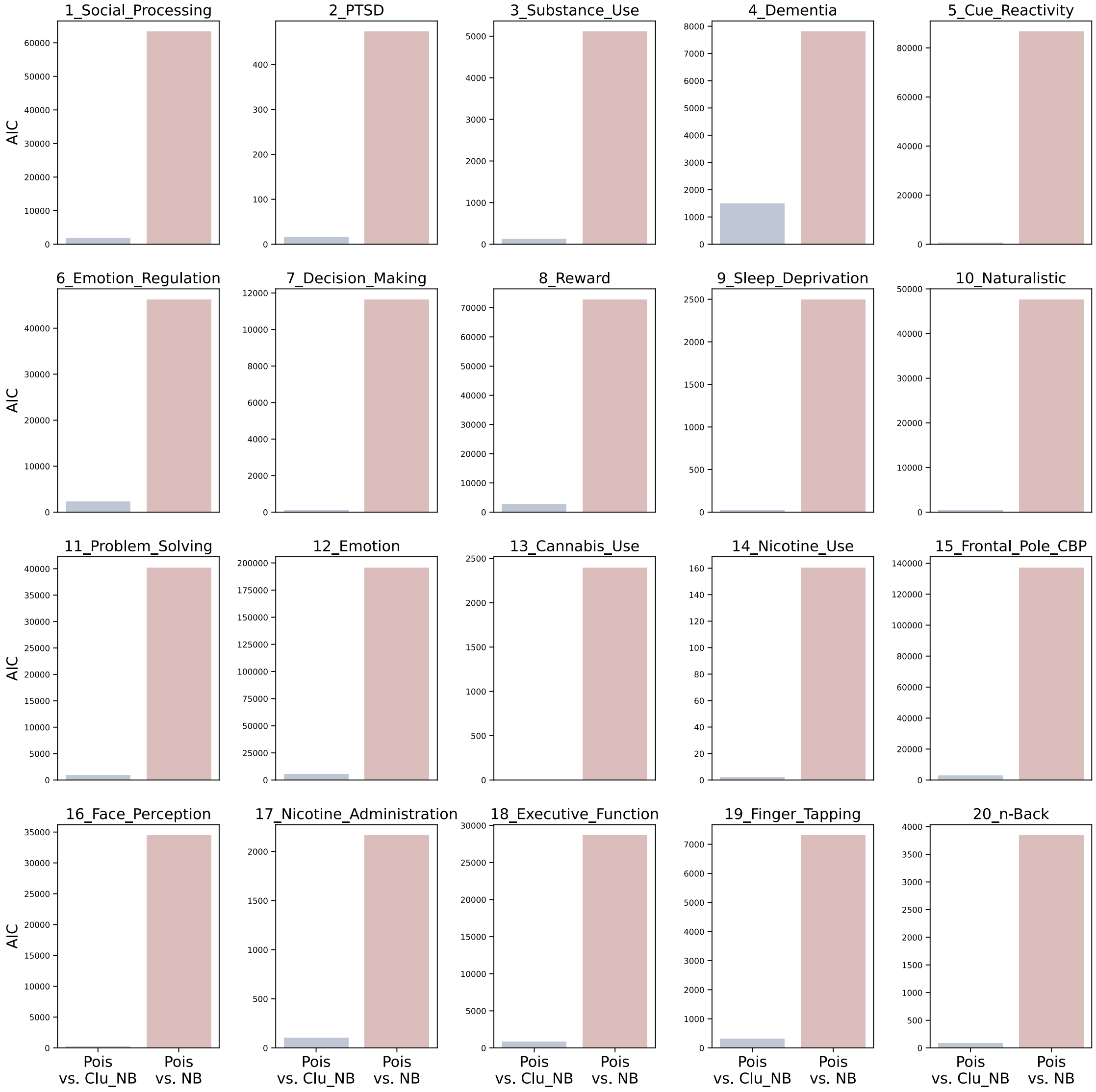

Figure S13: Likelihood-based comparison of CBMR with Poisson, NB and clustered NB models (difference in AIC, with Poisson model as the reference). We found that the AIC of NB model is always the smallest, while for some datasets, the difference of AIC between clustered NB and Poisson model is negligible, therefore, the least information loss is observed in NB model.

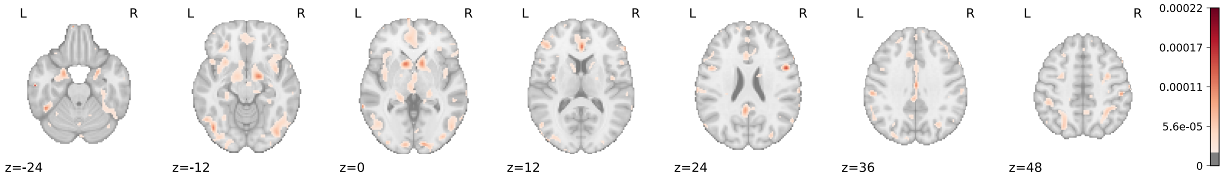

(a) Estimated Intensity for the Cue Reactivity Dataset Using CBMR with a NB Model

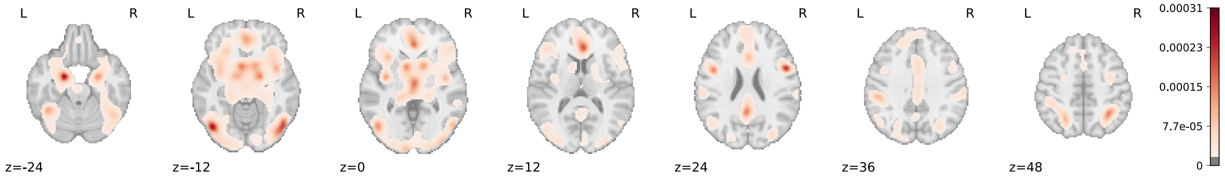

(b) Estimated Intensity for the Cue Reactivity Dataset Using Bayesian LGCP approach

Figure S14: Estimated intensity maps for the Cue Reactivity Dataset with CBMR (with NB model) and LGCP approaches
